# The human OPA1^delTTAG^ mutation induces adult onset and progressive auditory neuropathy in mice

**DOI:** 10.1007/s00018-024-05115-4

**Published:** 2024-02-09

**Authors:** Corentin Affortit, Carolanne Coyat, Anissa Rym Saidia, Jean-Charles Ceccato, Majida Charif, Emmanuelle Sarzi, Frédéric Flamant, Romain Guyot, Chantal Cazevieille, Jean-Luc Puel, Guy Lenaers, Jing Wang

**Affiliations:** 1 Institute for Neurosciences of Montpellier (INM), University Montpellier, INSERM, UMR 1298, 80 Rue Augustin Fliche, 34295 Montpellier, France; 2 Genetics, and Immuno-Cell Therapy Team, Mohamed First University, 60000 Oujda, Morocco; 3 Institut NeuroMyoGène, Pathophysiology and Genetics of Neuron and Muscle (INMG-PGNM) UCBL-CNRS UMR5261, Inserm U1315, Université Claude Bernard, Lyon I, Faculty of Medicine and Pharmacy, Lyon, France; 4 Institut de Génomique Fonctionnelle de Lyon (IGFL), INRAE USC1370, CNRS (UMR5242), ENS Lyon, Lyon, France; 5 Université Angers, MitoLab Team, Unité MitoVasc, UMR CNRS 6015, INSERM U1083, SFR ICAT, Angers, France; 6 Service de Neurologie, CHU d’Angers, Angers, France; 7 Department of ENT and Head and Neck Surgery, University Hospital of Montpellier, Montpellier, France; 8 Molecular Otolaryngology and Renal Research Laboratories, Department of Otolaryngology, Head and Neck Surgery, University of Iowa, Iowa City, IA 52242 USA

**Keywords:** Hereditary optic neuropathy, Inner ear, Inner hair cell, Outer hair cell, Retina, Deafness, Hidden hearing loss, Mitochondrial homeostasis

## Abstract

Dominant optic atrophy (DOA) is one of the most prevalent forms of hereditary optic neuropathies and is mainly caused by heterozygous variants in *OPA1*, encoding a mitochondrial dynamin-related large GTPase. The clinical spectrum of DOA has been extended to a wide variety of syndromic presentations, called DOA*plus*, including deafness as the main secondary symptom associated to vision impairment. To date, the pathophysiological mechanisms underlying the deafness in DOA remain unknown. To gain insights into the process leading to hearing impairment, we have analyzed the *Opa1*^*delTTAG*^ mouse model that recapitulates the DOA*plus* syndrome through complementary approaches combining morpho-physiology, biochemistry, and cellular and molecular biology. We found that *Opa1*^delTTAG^ mutation leads an adult-onset progressive auditory neuropathy in mice, as attested by the auditory brainstem response threshold shift over time. However, the mutant mice harbored larger otoacoustic emissions in comparison to wild-type littermates, whereas the endocochlear potential, which is a proxy for the functional state of the *stria vascularis*, was comparable between both genotypes. Ultrastructural examination of the mutant mice revealed a selective loss of sensory inner hair cells, together with a progressive degeneration of the axons and myelin sheaths of the afferent terminals of the spiral ganglion neurons, supporting an auditory neuropathy spectrum disorder (ANSD). Molecular assessment of cochlea demonstrated a reduction of *Opa1* mRNA level by greater than 40%, supporting haploinsufficiency as the disease mechanism. In addition, we evidenced an early increase in Sirtuin 3 level and in Beclin1 activity, and subsequently an age-related mtDNA depletion, increased oxidative stress, mitophagy as well as an impaired autophagic flux. Together, these results support a novel role for OPA1 in the maintenance of inner hair cells and auditory neural structures, addressing new challenges for the exploration and treatment of OPA1-linked ANSD in patients.

**Supplementary Information:**

The online version contains supplementary material available at 10.1007/s00018-024-05115-4.

## Introduction

Dominant optic atrophy (DOA) is the most prevalent form of hereditary optic neuropathy [1] with a frequency of 1:20 000, and is caused mainly by heterozygous variants in *OPA1,* encoding an ubiquitous mitochondrial dynamin large GTPase [2–4]. OPA1 is involved in many mitochondrial functions, notably in the maintenance of the respiratory chain and membrane potential [5–7], cristae organization, control of apoptosis [6, 8, 9], mitochondrial DNA (mtDNA) maintenance [10–13], and mitochondrial homeostasis [14–16]. DOA has been initially described as a non-syndromic moderate to severe loss of visual acuity with an insidious onset in early childhood, caused by a progressive and selective loss of retinal ganglion cells [17]. In the last decade, DOA clinical spectrum related to *OPA1* variants has been extended to a wide variety of symptoms called DOA*plus*, combining deafness, ataxia, neuropathy, myopathy, Parkinsonism, and dementia [10, 18, 19]. Deafness is the second most prevalent clinical feature in DOA*plus*, affecting about 20% of all DOA patients [10, 18–23].

The association of DOA and deafness is frequently related to the c.1334G > A variant (p.R445H) in exon 14, but other *OPA1* missense variants were reported in the literature [18, 24]. Hearing loss starts in childhood or early adulthood [18, 25]. Although the majority of studies qualified the hearing disorder as sensorineural hearing loss, some authors have proposed auditory neuropathy as the pathophysiological mechanism underlying the hearing impairment in DOA*plus* [5, 20, 26–28].

Auditory neuropathy, first introduced by Starr et al. [29], is a form of hearing disorder in which the auditory brainstem responses (ABRs) reflecting the synchronous activation of the relays along the ascending auditory pathway are absent or desynchronized. By contrast, the oto-acoustic emissions corresponding to the mechanical activity of the outer hair cells (OHC), which amplify the sound stimulation within the cochlea, are preserved [29]. Indeed, the inner hair cells (IHCs) transduce the acoustic cues into a receptor potential which in turn governs the release of transmitter onto the afferent terminals of the spiral ganglion neurons (SGNs), which convey the neural message through spike rate up to the cochlear nuclei [27, 29–31]. Currently, the pathophysiology of the auditory disorders in DOA*plus* is still poorly understood, and no curative treatment is available for *OPA1*-related degeneration of the optic, auditory, and other nerves.

To gain insights into the pathophysiology of hearing impairments linked to *OPA1* mutation, we have studied the heterozygous *OPA1*^delTTAG^ mouse model, which recapitulates the DOA*plus* syndrome [32]. Interestingly, in addition to finding that this mutation induces an adult-onset progressive hearing loss concomitant with IHCs loss, reduction of terminal dendrites, degeneration of ganglion cell somas and Schwann cells, we disclose here that Opa1 function is required to maintain the IHC and auditory neural structures during the aging process.

## Materials and methods

To decipher the mechanisms responsible for the auditory deficits in DOA, we used a transgenic heterozygous mouse model carrying a human recurrent *OPA1*^delTTAG^ mutation recapitulating the DOA*plus* syndrome. We first determined the consequence of this mutation on cochlear anatomy and physiology. Then, we identified the underlying mechanisms mediating the degeneration of the cochlear cells associated with DOA.

### Animals

The generation of *Opa1* knock-in mouse in a mixed C57BL/6 J × 129 Sv/Pas genetic background carrying the recurrent human *OPA1* c.2708_2711delTTAG mutation has yet been described [32]. *Opa1* mouse breeding generated only WT and heterozygous Opa1 (*Opa1*^±^) mice, because homozygous mice dye in utero before embryonic stage 10.5. *Opa1*^±^ mice developed normally over time with no significant reduction of the lifespan observed when compared with WT mice [32]. A previous report showed that female *Opa1*^±^ mice exhibited earlier onset and exacerbated vision loss and retinal ganglion cell degeneration than males, due to high circulating levels of steroid precursor pregnenolone and altered expression of estrogen receptors in female mice [33]. Therefore, in this study, we investigated both male and female *Opa1*^±^and their littermate control (WT). To study the effects of *Opa1* mutation in hearing maturation and maintenance of cochlear cells during adulthood in mice, functional, morphological and molecular evaluations were performed in juvenile mice (1-month-old) and then every 3 or 6 months until 12 months. Later at 18 months, mutant mice display total hearing loss, while WT mice were profoundly deaf, as already reported in the literature for age-related hearing loss in mice with the mixed genetic background 129/SV and C57BL/6 [34]. Mice were housed in a facility core accredited by the French Ministry of Agriculture and Food (D-34 172 36—19 December 2020). 

### Genotyping

Genotyping was performed using routine PCR with the forward primer 5ʹ-GGA GGA TGT GTG TAT AGC ATA GCC ATT GG-3ʹ and reverse primer 5ʹ-CAA AAC CAC CAA GTA GTG CTC AGG ACG-3ʹ, to amplify genomic DNA extracted from tail tips of mice. The resulting PCR products, 795 bp from the WT allele and 935 bp from the delTTAG allele, were resolved on a 2% agarose gel and visualized by ethidium bromide staining. All primers were synthesized by Eurofins MWG Operon. PCR amplification was performed using the following protocol: 95 °C for 3 min, 35 cycles (95 °C for 15 s, 65 °C for 15 s and 72 °C for 60 s), and 72 °C for 5 min.

### Auditory functional assessments

All functional evaluations were performed in mice anesthetized by an intraperitoneal injection of Rompun 2% (3 mg/kg) and Zoletil 100 (40 mg/kg). Recordings were performed in male and female *Opa1*^±^ mice and wild-type counterparts. 25 mice of each strain were successful monitored by auditory brainstem response (ABR) and distortion product otoacoustic emission (DPOAE) assessments up to 12 months of age. Among them, 10 were randomly selected at 12 months of age for endocochlear potential (EP) recording and 15 for compound action potential (CAPs) recordings and sacrificed for cochlear morphological and molecular assessments. 20 additional animals aged 1 and 6 months (*n* = 10 per age and per strain) were needed for EP recording (Table S1). After recording ABR, DPOAE, and EP, cochleae were removed for morphological and molecular evaluations. All functional evaluations were carried out in a Faraday-shielded, anechoic, sound-proof cage. Rectal temperature was measured with a thermistor probe and maintained at 38.5 °C ± 1 using an underlying, heated blanket.

#### Distortion product otoacoustic emissions

DPOAEs were recorded in the external auditory canal using an ER-10C S/N 2528 probe (Etymotic research Inc. Elk Grove Village, IL, USA.). The two primary frequency tones f1 and f2 with a constant f2/f1 ratio of 1.2. were generated, and the distortion product 2f1-f2 processed, by a Cubdis system HID 40133DP (Mimosa Acoustics Inc., Champaign, IL, USA). The probe was self-calibrated for the two stimulating tones before each recording. f1 and f2 were presented simultaneously, sweeping f2 from 20 to 2 kHz by quarter octave steps. For each frequency, the distortion product 2f1-f2 and the neighboring noise amplitude levels were measured and expressed as a function of f2.

#### Auditory brainstem responses

ABRs were recorded using three subcutaneous needle electrodes placed on the vertex (active), on the pinna of the tested ear (reference), and in the hind leg (ground). The acoustic stimuli generated by a NI PXI-4461 signal generator (National Instruments) consisted of 10 ms tone bursts with a 1 ms rise- and fall time, delivered at a rate of 10/s. Sound was produced by a JBL 075 loudspeaker (James B. Lansing Sound) positioned at 10 cm from the tested ear in a calibrated, free-field condition. Cochlear responses were amplified (20,000) via a Grass P511 differential amplifier, and averaged 1000 times (Dell Dimensions). Amplitude-intensity functions of the ABRs were obtained at each frequency tested (2, 4, 6.3, 8, 10, 12.5, 16, 20, 25, and 32 kHz) by varying the level of the tone bursts from 0 to 100 dB SPL, in 5 dB incremental steps. ABR thresholds were defined as the minimum sound intensity necessary to elicit well-defined and reproducible wave-II. Recordings and analysis were performed blindly.

#### Compound action potential of the auditory nerve

To record the CAP, which is the result of synchronous activity of the auditory nerve fibers in response to the sound stimulus, a retroauricular skin incision was made to access to the tympanic bulla, which was then opened to expose the round window. A silver electrode placed on the round window recorded the CAP evoked by tone bursts (9 ms duration, 1 ms rise/fall, 10/s). CAP thresholds (2, 4, 6, 8, 10, 12, 16, 20, 26, and 32 kHz) were defined as the minimum sound intensity necessary to elicit a clearly distinguishable response [35].

#### Endocochlear potential

To measure the EP, which is a proxy for the functional state of the *stria vascularis*, the bone of the *scala media* basal turn was gently shaved off, resulting in a small fenestra. A glass microelectrode (tip diameter 0.1–0.5 µm), filled with 0.15 M KCl and connected to a direct current amplifier (WPI, model 773 A; Sarasota, FL, USA), was placed visually at a position and angle allowing it to pass through the fenestra, to record the EP with reference to an Ag/AgCl reference electrode in the animal neck musculature.

### Sensory hair cell morphological evaluation and counting

Morphological evaluation and counting of sensory hair cell were performed with a scanning electron microscopy (SEM, Hitachi S4000). Cochleae from WT and mutant mice aged 1, 3, 6, and 12 months (*n* = 6 to 8 cochleae per age and genotype, see Table S1) were processed and evaluated using previously reported standard techniques [36–38]. Hair cell counting was performed in apical (0.1 to 1 mm from the apex tip, corresponding to the 4 to 8 kHz region), mid (1 to 2.5 mm from the apex tip, corresponding to the 8 to 16 kHz region), and basal (2.5 to 4 mm from the apex tip, corresponding to 16–32 kHz region) regions of the cochlea. Hair cells were considered absent if the stereociliary bundles and cuticular plates were missing.

#### Auditory nerve terminal counting 

The density of the auditory nerve fiber terminals was measured in 3 to 4 habenular openings of cochlear semi-thin sections of the osseous spiral from the cochlear regions coding between 16 and 32 kHz of WT and mutant mice aged 1, 3, 6, and 12 months. The mean value of each section was then averaged for each animal and each group (*n* = 3 sections per animal, 5–6 cochleae per age and genotype, Table S1). The measurement of auditory nerve fiber size and myelin thickness was performed using custom interface written in Matlab (Mathworks). Using manually driven thresholding on transmission electron microscopy (TEM, Tecnai F20 FEI 120 kV) micrographs, auditory nerve fibers were automatically labeled as region of interest (ROI). After manual validation of each detected ROIs, myelin was detected using “balloon inflation” algorithm (*imdilate* function) from ROIs until reaching the inter-cells region. Size and area of items were determined from the number of pixels of the ROI, by deducing respectively the side length (micrometers) and area (square micrometers) per pixel from the scale bar.

#### Counting of spiral ganglion neurons

The SGN density in Rosenthal’s canal was measured using a Zeiss Axioskop light microscope in semi-thin sections that had been cut during the course of TEM preparation and stained with 1% toluidine blue. The SGN counts were calculated in the basal region of the cochlea. NIH Image J software was used to determine the cross-sectional area of Rosenthal’s canal. SGN density was calculated by dividing the number of neurons by the cross-sectional area (*n* = 5 sections per cochlea, 4 to 5 cochleae per age and strain, Table S1).

#### Transmission electron microscopy and histological analysis

Morphological damage was investigated by TEM. Since statistical analysis had been performed on 16–32 kHz responses, TEM investigations focused on the upper half of the cochlear basal turn (*n* = 5–6 cochleae per age and genotype). Animals were decapitated under deep anesthesia, and their cochleae were prepared according to a standard protocol for fixation and plastic embedding. Semi-thin sections cut during the course of TEM preparation were observed under a Zeiss Axioskop light microscope and ultrathin radial sections of the organ of Corti were observed using TEM. All affected fibers carrying electron-dense bodies, autophagic vacuoles, aberrant myelin features as well as the number of mitochondria per axon were quantified in cross sections of auditory nerve fiber terminals on electron microscopy pictures (*n* = 3 sections per animal, 5–6 cochleae per age and genotype, Table S1).

### RNA seq

RNA was extracted from isolated cochlea using TRI Reagent (Sigma-Aldrich, Life Science), further purified on NucleoSpin microcolumns (Macherey–Nagel). RNAs were quantified with Qubit (ThermoFisher Scientific), and controlled by microelectrophoresis (TapeStation 4200, Agilent). cDNA libraries were prepared using the CORALL RNA seq Library Prep Kit (Lexogen). Paired-end sequencing (2 × 80 nucleotides) was performed using a NextSeq500 sequencer (Illumina) and raw data analyzed using the CORALL Data Analysis Pipeline. Demultiplexing was performed using the unique molecular identifiers included in the adapters to correct for possible amplification biases. Sequence reads passing the quality control (fastQC; %Q30 > 93.) were trimmed from adapter sequences and mapped to the mouse genome (GRCm38/mm10 version Dec 2011) using the STAR aligner. The number of mapped reads was between 21 × 10^6^ and 27 × 10^6^. Deseq2 was used to determine differentially expressed genes from count tables (Galaxy version 2.11.40.6 + galaxy2; threshold: basemean > 10, adjusted P < 0.05). RNAseq required 4 *Opa1*^**±**^ mice and 4 control littermates at post-natal p21. RT-qPCR was performed on mRNA extracts from cochlear tissues of 5 *Opa1*^±^ and 5 control samples with iQ SyBR Green Supermix (Biorad) according to the manufacturer’s instructions. All experiments were performed with biological and technical triplicates, and results were normalized by HPRT. Raw data and count tables are accessible in the Gene Expression Omnibus (GEO) repository (accession number: GSE232051).

### MtDNA sequencing and analysis

The entire mtDNA molecule was amplified with two over-lapping 8.9 (7036-15990) and 9.3 (14732-7447) kilo bases (kb) fragments respectively. Library preparation was performed using the Ion Plus Fragment Library Kit (Cat. no. 4471269). Sample emulsion PCR, emulsion breaking, and enrichment were performed using the Ion 540 Kit–Chef (Cat. No. A27759) and sequenced on the Ion S5 Sequencer. Sequencing data base calling and mapping were performed using Ion Torrent Suite. The variant calling module uses a consensus-based approach and the prediction with GATK Unified Genotyper [39] caller. All the generated variants were analyzed and confirmed with IGV software and Blast tool.

Searching for mtDNA deletions and insertions was performed using the eKLIPSE program, which is based on a soft-clipping analysis [40]. A comparison between both groups was carried out using the non-parametric test (Mann–Whitney test). MtDNA sequencing needed 5 mice per age and per genotype (Table S1).

### Quantification of mtDNA copy number

For mtDNA quantification, total DNA was isolated from cochleae (*n* = 5 mice per age and genotype) using the qiagen “QIAamp^®^ DNA Mini Kit” according to the protocol provided. The mitochondrial COX1 gene was amplified together with the nuclear NDUFV1 gene as a normalizing control. Primers were COX1: F-5′-TGCTAGCCGCAGGCATTAC -3′ and R-5′-GGGTGCCCAAAGAATCAGAAC -3′ and NDUFV1: F-5′-CCCCACTGGCCTCAAG-3′ and R-5′-CCAAAACCCAGTGATCCAGC-3′. QPCRs were performed in triplicate in 96-well reaction plates as described elsewhere [41].

### Oxidative stress

Cochlear homogenates were prepared as described [42]. The protein concentration was measured using the Bradford method. Catalase activity was measured as previously described [43]. The concentration of thiol levels in total extracts from Cochlear homogenates was measured as described [44]. Oxidative stress analyses required 8 additional animals (16 cochleae) per age and strain (Table S1). All experiments were performed in triplicate.

### mRNA levels of mitochondrial fission genes

mRNA was extracted from 8 cochleae per sample (Table S1) using Trizol method. Samples were reverse transcribed using PrimeScrip RT Reagent Kit (Takara). Quantitative PCR reactions were performed with FAST SYBR master mix (ROCHE) according to the manufacturer’s instructions. PCR analysis required 8 cochleae per condition. All experiments were performed in biological and technical triplicate, results were normalized to β-actin mRNA. Primers were Dnm1l: F-TCAGATCGTCGTAGTGGGAA and R-TCTTCTGGTGAAACGTGGAC and Mfn1: F-CCAGGTACAGATGTCACCACAG and R-CCAGGTACAGATGTCACCACAG. qPCRs were performed in triplicate in 96-well reaction plates as described elsewhere.

### Immunoblotting

Cochlear homogenates were prepared in Laemmli sample buffer. Blots were incubated with antibodies recognizing Sirtuin 3 (SIRT3, 1/1000, Cell Signaling #5490 RRID:AB_10828246), p-Beclin 1 (1/200, Cell Signaling Ser15, # 84966 RRID:AB_2800045), Nrf2 (1/1000, Santa Cruz Biotechnology # sc-365949, RRID:AB_10917561), SOD2 (1/1000, abcam #ab13533, RRID:AB_300434), Catalase (1/1000, sigma Aldrich #SAB4503383, RRID:AB_10747206), LC3B (1/800, Cell Signaling #2775 RRID: AB-915950), Bnip3 (1/1000, Abcam, #Ab10433 RRID:AB-2066656), Parkin (1/1000, Santa Cruz Biotechnology #sc-32282, RRID:AB_628104), Rab7 (1/800, Santa Cruz Biotechnology #sc-376362, RRID:AB-10987863), and Bax (1/1000, Abcam #ab7977, RRID:AB-306191). β-actin (1/10000, Sigma-Aldrich #A1978, RRID:AB-476692) was used as a loading control. Secondary antibodies were horseradish peroxidase-conjugated goat anti-mouse IgG (1/3000, Jackson ImmunoResearch #115-001-003, RRID: AB-2338443) or goat anti-rabbit IgG (1/3000, Jackson ImmunoResearch #111-001-003, RRID: AB-2337910). Image scans of Western blots were used for semi-quantitative analysis. Western blot analysis required 24 cochleae per age and genotype (Table S1). Each experiment with a pool of 8 cochleae was performed in biological and technical triplicate. All results were normalized by β-actin expression.

### Immunocytochemistry

Immunocytochemistry was used to probe the cellular localization of Opa1, Cytochrome c oxidase (Cox) and some autophagic and antioxidant markers in cryostat sections using antibodies recognizing Opa1 (1/125; Abcam #ab42364 RRID:AB_944549)**,** Cytochrome c oxidase subunit I (1/500; Invitrogen, #459600 RRID:AB-1501840), p62 (1/1000, MBL International #PM045 RRID:AB_1279301) and Nrf2 (1/1000, Santa Cruz Biotechnology # sc-365949 RRID:AB_10917561). Anti-parvalbumin (1/500; Swant, Bellinzona, Switzerland, #PV235 RRID:AB_10000343) was used to label the hair cells and the spiral ganglion neurons. All secondary antibodies were used at a dilution of 1/1000. This included donkey anti-mouse and anti-rabbit IgG conjugated to Alexa 488 or Alexa 568 (Molecular Probes #A-21202 RRID:AB-141607, #A-21206 RRID:AB-2535792, #A-10037 RRID:AB-2534013). DNA was stained by Hoechst 33342 (0.002% wt:vol, Sigma, Saint Louis, Missouri, USA). Fluorescent tags were visualized using a confocal microscope (ZEISS LSM 880 Airyscan**)**. In control specimens without primary antibodies, neither Alexa 488 nor 568 fluorescent tags were observed. Immunocytochemistry analysis required 4 to 5 cochleae per age and strain (Table S1). All experiments were performed in triplicate.

### P62 immunodensity

The semiquantitative analysis of p62 green immunofluorescence was analyzed using ImageJ software in transverse sections of Rosenthal’s canal of cochlear basal regions from both WT and *Opa1*^±^ mice aged 1 and 6 months. The fluorescence intensity was measured in the somas of SGNs. For each cochlea, ~ 30 neurons, were taken randomly from 2 cochlear sections (*n* = 3 cochleae per age and genotype).

### Statistics

Data are expressed as mean ± SEM. Normality of the variables was assessed using the Shapiro–Wilk test. The significance of the group differences was assessed with a one-way ANOVA; once the significance of the group differences (*P* ≤ 0.05) was established, Dunn’s tests were used for post hoc comparisons between pairs of groups. The *P* values are indicated in the legends for each figure. Based on data from our previous reports [45] or from preliminary experiments, we calculated the sample size using G*Power 3.1.9.2 to ensure adequate power of key experiments for detecting pre-specified effect sizes.

## Results

### *Opa1*^+/-^ mice show exacerbated age-related hearing loss

To determine the functional effect of *Opa1*^delTTAG^ mutation on hearing maintenance during aging, we assessed the auditory function by recording sound-evoked CAPs and ABRs, which reflect the synchronous activation of auditory neurons from the cochlea up to the colliculi in response to incoming sound. We found that WT mice displayed a classic high-frequency age-related hearing loss (ARHL) as early as 6 months in male mice and at 12 months in female mice (Fig. S1A). The average ABR thresholds at 32 kHz were significantly increased in male mice aged 3 months (*P* < 0.05), and onwards to 12 months, while significant increase was only observed in 12-month-old WT female mice (*P* < 0.001, Fig. S1B). At 12 months, both male and female displayed similar elevated thresholds (mean thresholds of 64 ± 5.9 and 73 ± 3.7 dB SPL for males and females, respectively, Fig. S1B). The same ARHL tendency for both sexes was observed in *Opa1*^±^ mice (Fig. S1C). At 12 months, male and female *Opa1*^±^ mice exhibited identical elevated thresholds (mean thresholds of 80 ± 4.2 and 81 ± 4.2 dB SPL at 32 kHz for males and females, respectively, Fig. S1D). The relative hearing preservation observed in both WT and mutant female mice at 3 to 6 months of age (Fig. S1A–D) is explained by a protective effect from estrogens against ARHL as reported in previous studies [46].

**Fig. 1:**
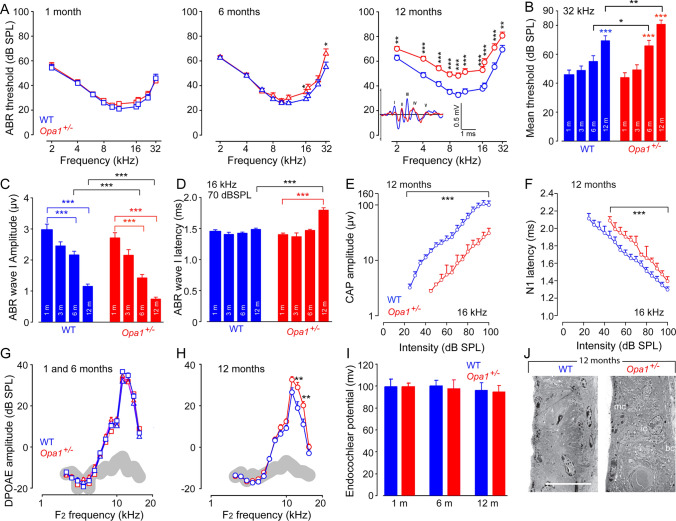
*Opa1*^*delTTAG*^ mutation leads to exacerbated age-related hearing loss. **A** Auditory brainstem response (ABR) thresholds recorded in WT and *Opa1*^**±**^ mice aged 1, 6 and 12 months. (**Insert** in **A**) mean ABR waveforms evoked by 16-kHz tone bursts at 70-dB sound pressure level (SPL). **B** Mean ABR thresholds at 32 kHz recorded in in WT and *Opa1*^**±**^ mice aged 1, 3, 6 and 12 months. **C** and** D** Mean wave-I amplitude (**C**) and latency (**D**) evoked by 16 kHz tone bursts at 70 dB sound pressure level (SPL) in WT and *Opa1*^**±**^ mice aged 1, 3, 6 and 12 months. **E** and **F** Shown are the input–output functions of the compound action potential (CAP) and N_1_ latency evoked by 16 kHz tone bursts in WT and *Opa1*^**±**^ mice aged 12 months. **G** and** H** Distortion product otoacoustic emission (DPOAE) amplitudes recorded in WT and *Opa1*^**±**^ mice aged 1 and 6 (**G**), and 12 months (**H**). **I** Measurements of the endocochlear potential magnitude in WT and *Opa1*^**±**^ mice aged 1, 6 and 12 months. All data are expressed as mean ± SEM (*n* = 25–40 mice per genotype and time point for ABR and DPOAE recording, *n* = 10 and 15 for EP and CAP recording, respectively), one-way ANOVA test was followed by *Dunn’s* test: *P ≤ 0.05, **P ≤ 0.01, ***P ≤ 0.001. Black asterisks, *Opa1*^**±**^ vs. WT mice of the same age; red asterisks, older *Opa1*^**±**^ vs. 1-month-old *Opa1*^**±**^; blue asterisks, older WT vs. 1-month-old WT. **J** Representative micrographs of transmission electron microscopy showing that the stria vascularis has a normal appearance in both WT and *Opa1*^**±**^ mice at 12 months (MC: marginal cells, IMC: intermediate cells, BC: basal cells). Scale bar: 25 µm

Our results demonstrated that hearing thresholds are similar in males and females aged 1 and 12 months, both in mutant and WT genotypes. Additionally, comparable hearing preservation was observed in female WT and mutant mice aged 3 and 6 months. Together, these results suggest that the OPA1 mutation has no effect on the potential protective effect of estrogen in the cochlea, at least in mice. Therefore, in the following sections, results were obtained for both sexes (half male, half female). The combination of the data obtained from both sexes showed that ABR thresholds in *Opa1*^**±**^ were virtually identical to WT at 1 month of age (Fig. 1A). At later ages, both strains showed the progressive typical age-related increase of ABR thresholds (Fig. S2A), beginning at high frequencies and progressing toward low frequencies (Fig. 1A). Significantly higher ABR thresholds were observed at higher frequencies in 6- month-old *Opa1*^**±**^ mice and at all the frequencies tested in 12-month-old *Opa1*^**±**^ mice, compared to WT animals of the same age (*P* < 0.01, Fig. 1A). Mean ABR thresholds at 32 kHz were significantly higher in *Opa1*^**±**^ mice from the age of 6 months and maintained to 12 months compared to their WT littermates of the same age (Fig. 1B, *P* < 0.05).

### *Opa1*^±^ mice exhibit enhanced age-related changes in auditory brainstem responses

The amplitude of the ABR wave-I, capturing the synchronous activity of auditory nerve fibers, can be used as an objective measure of the loss of function of IHC ribbon synapses when measured at high sound intensity above 70 dB sound pressure level (SPL). Age-related decrease in ABR wave-I amplitudes elicited by 16 kHz tone bursts at 70 dB SPL was found in both strains from 3 months of age. This reduction in ABR wave-I amplitudes reached significance at 6 months in both genotypes and onwards to 12 months, but more importantly in *Opa1*^**±**^ mice (*P* < 0.001, vs. WT of the same age, Fig. 1C), indicating a decreased number of auditory nerve fibers activated by sound and/or a decrease in their synchrony. In addition, a significant increase in the latency of waves-I was also observed in *Opa1*^**±**^ mice aged 12 months compared with 1-month-old mutant mice or with WT mice of the same age (*P* < 0.001, Fig. 1D and insert in 1A). The measure of the CAP disclosed significant decrease in CAP amplitude and an increase in N1 latency of CAP elicited by 16 kHz tone bursts at all sound levels tested in 12-month-old *Opa1*^±^ mice, compared to age-matched WT mice (*P* < 0.001, Fig. 1E, F).

### *Opa1*^±^ mice have enhanced distortion product otoacoustic emissions and preserved endocochlear potential

OHCs act as nonlinear feedback amplifiers that enhance the sensitivity and the frequency selectivity of the hearing organ. Distortion product otoacoustic emissions (DPOAEs) are the by-product of this nonlinear amplification process and hence can serve as a measure for evaluating integrity of the OHCs. Our results showed that the amplitude of DPOAEs was preserved until 6-month age in both *Opa1*^**±**^ and WT mice (Fig. 1G). An age-related reduction in DPOAE amplitudes was observed in the frequency range of 10–20 kHz in 12-month-old WT mice (Fig. 1H). Conversely, DPOAE amplitude was maintained in 12-month-old *Opa1*^±^ mice leading to a significant difference between the two genotypes at the frequency of 10 and 16 kHz (*P* < 0.01, Fig. 1H, S2B). In addition, the endocochlear potential, which is a proxy for the functional state of the *stria vascularis*, was comparable between both genotypes (Fig. 1I). These functional results were then confirmed by TEM evaluation showing that no ultrastructural abnormality was detected in the stria vascularis of 12-month-old WT and *Opa1*^±^ mice (Fig. 1J).

Together, these data indicate that the heterozygous *Opa1*^*delTTAG*^ mutation accelerates natural age-related hearing loss, which is not caused by the impaired sound processing, nor to the dysfunction of the *stria vascularis* in *Opa1*^**±**^ cochlea, but most likely to a progressive alteration in the ascending auditory pathway affecting IHCs and auditory nerve fibers.

### *Opa1*^±^ mice display an early occurrence of selective degeneration and loss of inner hair cells

**Fig. 2:**
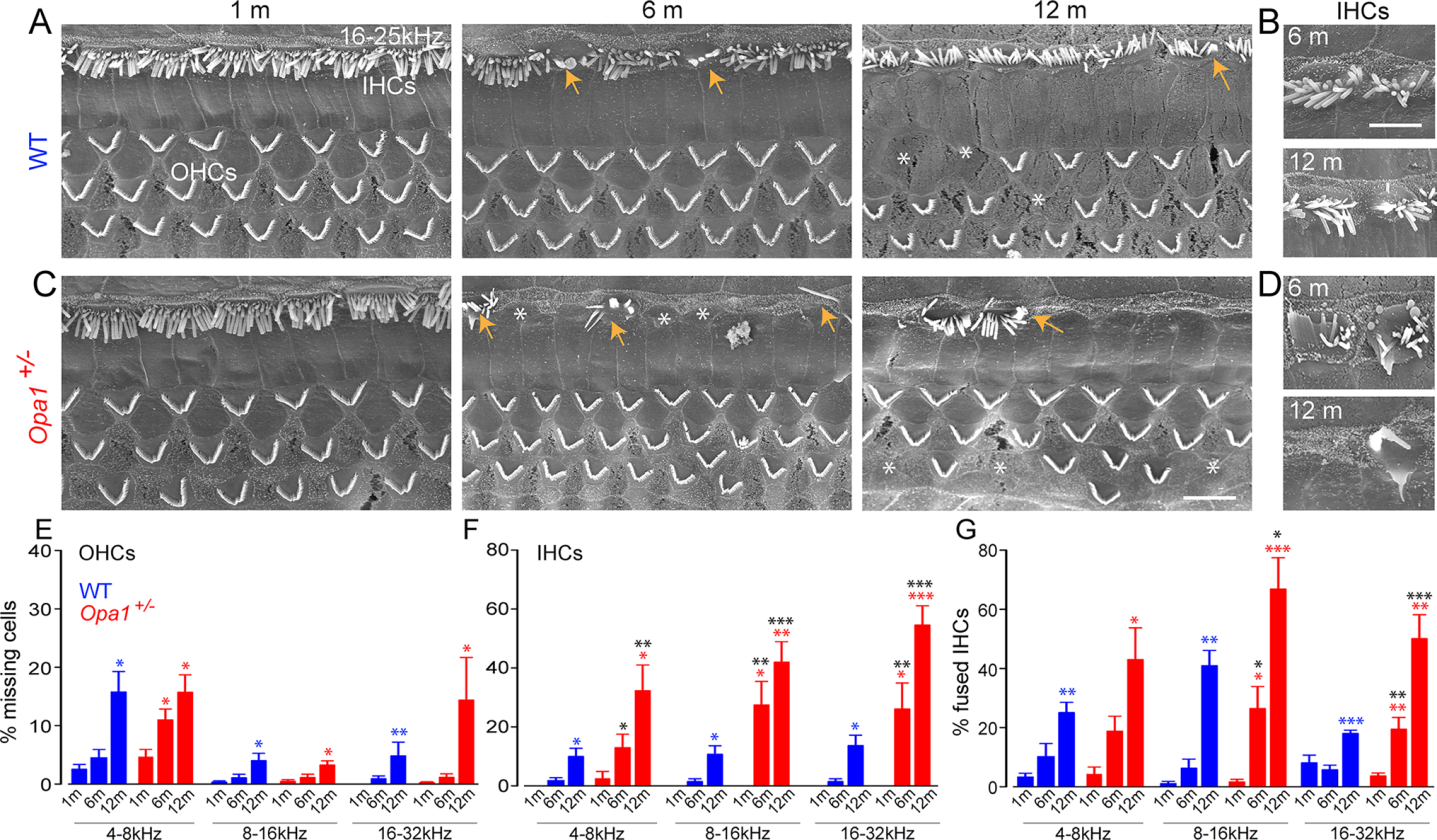
Selective loss of IHCs in *Opa1*^±^ mice. **A, C** Representative scanning electron microscopy from the cochlear regions coding 16-25 kHz from WT (**A**) and *Opa1*^±^ (**C**) mice aged 1, 6 and 12 months. **B**, **D** Higher magnification images of representative hair bundles of the IHCs from WT (**B**) and *Opa1*^±^ mice (**D**) at 6 and 12 months. Yellow arrows indicate fused hair bundles of the IHCs. White asterisks indicate missing IHCs and OHCs. Scale bars: **A, C** = 16 μm, **B, D** = 8 µm. **E–G** Histogram showing the percentage of missing OHCs (**E**) and IHCs (**F**), and IHCs with fused stereocilia bundles (**G**) in different coding regions (4–8, 8–16 and 16–32 kHz) from the cochleae of WT and *Opa1*^±^ mice aged 1, 6, and 12 months. Data are expressed as mean ± SEM (*n* = 6 to 8 cochleae per age and genotype). One-way ANOVA test was followed by *Dunn’s* test. *P ≤ 0.05, **P ≤ 0.01. Black asterisks: *Opa1*^±^ vs. WT mice of the same age, red asterisks: older *Opa1*^±^ vs. 1-month-old *Opa1*^±^, blue asterisks: older WT vs. 1-month-old WT

At 1 month of age, both WT and *Opa1*^**±**^ mice displayed a normal appearance of the surface morphology and the overall organization of the organ of Corti under SEM observation (Fig. 2A, C). At later stage, WT mice developed a slow and progressive age-related loss of OHCs, which became significant by 12-month of age (Fig. 2A, B), where some IHCs showed fused stereocilia throughout the cochlea, which was episodically observed from 6 months of age (Fig. 2A, B). In *Opa1*^**±**^ mice, massive selective IHC loss was observed as early as 6 months in whole cochlea (Fig. 2C, D), together with an increase in the fusion of stereocilia of the remaining IHCs throughout the cochlea (Fig. 2C, D). By contrast, age-related OHC loss in *Opa1*^**±**^ mice was chronologically similar to WT mice (Fig. 2A–D).

Counts of the sensory hair cells showed that both strains developed a slowly progressive age-related loss of OHCs, reaching significance at 12 months in the entire cochlea (Fig. 2E), except in the cochlear region coding 4–8 kHz, where a significant loss of OHCs was found in *Opa1*^**±**^ mice at 6 months (*P* ≤ 0.05 vs. 1 month, Fig. 2E). A significant increase (*P* ≤ 0.05) in IHC loss occurred only at 12 months of age in WT mice, while in *Opa1*^**±**^ mice, significant increase in IHC loss was observed at 6 months and onwards at 12 months in the whole cochlea (missing IHCs in basal region: 54.6% ± 6.5 for *Opa1*^**±**^ vs. 13.6% ± 3.5 for WT mice at 12 months, *P* ≤ 0.001, Fig. 2F). Similarly, a significantly increased IHC number with fused stereocilia was found at 12 months in WT mice whereas, in *Opa1*^**±**^ mice, this phenomenon was seen as early as 6 months in the cochlear regions coding 8 to 32 kHz (Fig. 2G). Then, at 12 months of age, IHC numbers with fused stereocilia were significantly higher than 1 month of age in whole cochlea of both strains (*P* ≤ 0.05), but being more severe in *Opa1*^**±**^ mice than in WT mice (fused stereocilia of IHCs in basal region: 50.1% ± 8.1 for *Opa1*^**±**^ vs. 17.9% ± 1.1 for WT mice, *P* ≤ 0.001, Fig. 2G). Together, these results indicate that selective IHC loss contributes to the acceleration of age-related hearing loss observed in *Opa1*^±^ mice.

### *OPA1*^*delTTAG*^ mutant mice displayed enhanced age-related loss and ultrastructural changes of the auditory nerve terminals and spiral ganglion neurons compared to WT mice

**Fig. 3:**
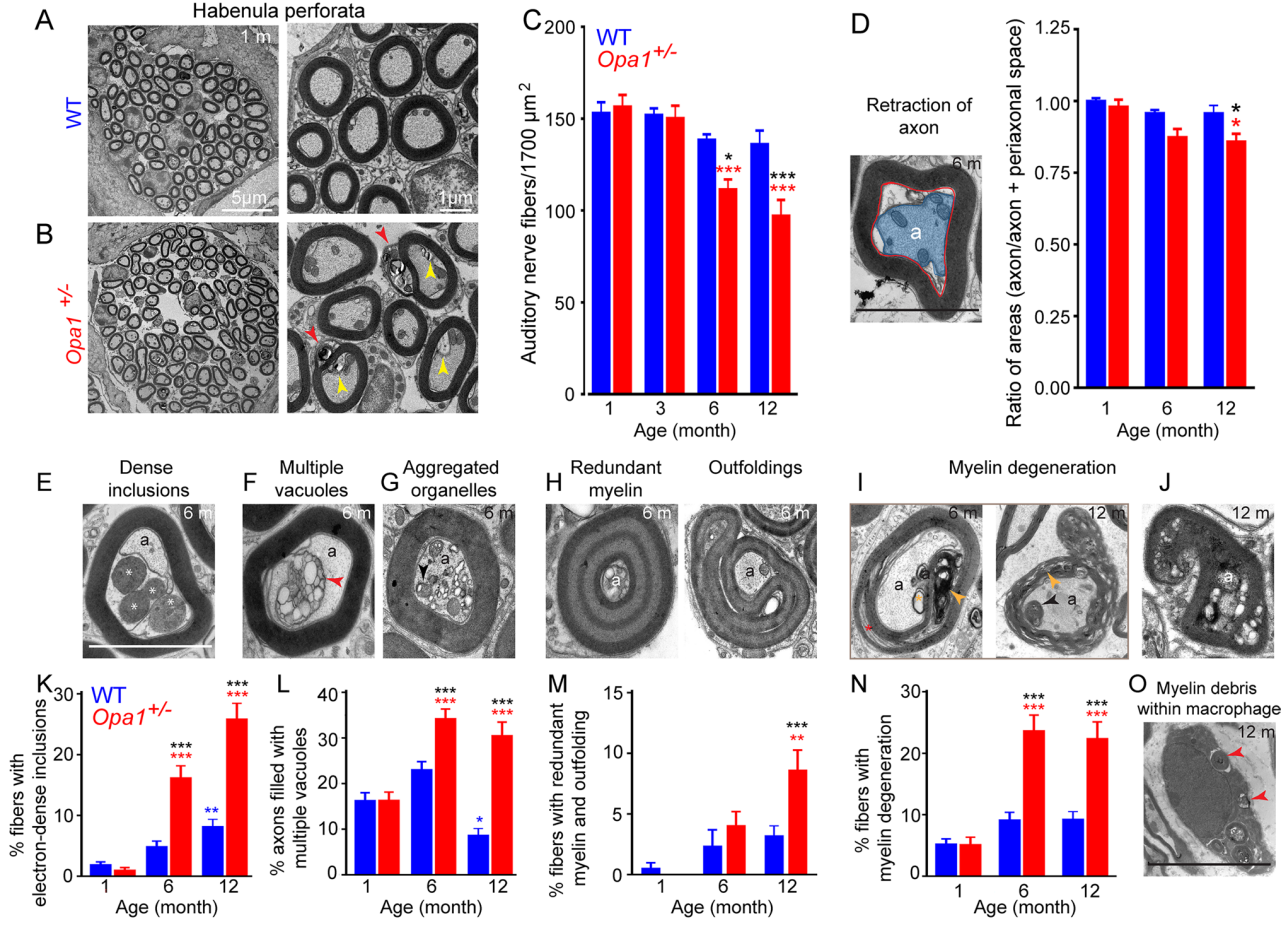
Alterations of the auditory nerve fibers in *Opa1*^±^ mice. **A** and** B** Representative micrographs of transmission electron microscopies showing the auditory nerve fibers (ANFs) in the habenula perforata from the upper basal turn of cochleae of WT (**A**) and *Opa1*^±^ (**B**) mice. Scale bars = 5 and 1 µm, respectively. **C** Quantitative assessment of ANF density in WT and *Opa1*^±^ mice aged 1, 3, 6, and 12 months (*n* = 3 sections per cochlea, 5 to 6 cochleae per age and genotype). Left in **D** Representative transmission electron micrograph of ANF from 6-month-old *Opa1*^±^ mice. The blue area indicates a retracted axon; the red line delimits the region occupied by the axon + periaxonal space. Scale bar** = **1 µm. Right in** D** Ratios of areas (axonal area/ axonal area + periaxonal space) from 200 and 400 individual fibers from 5 WT and 6 *Opa1*^±^ cochleae, respectively. **E–J** Representative transmission electron micrographs of ANFs from 6- and 12-month-old *Opa1*^±^ mice. Showing axons filled with electron dense inclusions (**E**), typical autophagic vacuoles (**F**), degraded organelles (**G**), ANFs with redundant myelin loops and outfoldings (**H**), split lamellae myelin and dense myelin debris (**I**), and degenerated ANF (**J**) (*n* = 3 sections per cochlea, 5 to 6 cochleae per age and genotype). Scale bar** = **1 µm. **K**–**N** Quantitative analysis of percentage of ANFs with electron dense bodies (**K**), axons filled with multiple vacuoles (**L**), ANFs with hypermyelination (**M**) and degenerating sheaths (**N**) (*n* = 200 and 400 individual fibers from 5 WT and 6 *Opa1*^±^ cochleae, respectively). All data are expressed as mean ± SEM, one-way ANOVA test was followed by *Dunn’s* test: ***P ≤ 0.001. Black asterisks: *Opa1*^±^ vs. WT mice of the same age, red asterisks: older *Opa1*^±^ vs. 1-month-old *Opa1*^±^*,* blue asterisks: older WT vs. 1-month-old WT. **O** Representative transmission electron micrograph shows a macrophage containing myelin debris. Scale bar = 2.5 µm

To determine the consequences of *Opa1*^delTTAG^ mutation on auditory nerve fibers (ANFs) and on SGNs, we compared the density and ultrastructure of the ANFs in the habenular openings in WT and *Opa1*^**±**^ mice (Fig. 3). At 1 month of age, both strains showed similar density of ANF terminals (153.0 ± 5.9 vs. 156.6 ± 6.4 ANFs per 1 700 µm^2^ for WT and *Opa1*^**±**^ mice, respectively, Fig. 3A–C), but some anomalies, such as vacuoles, electron dense deposits, and damaged mitochondrial remnants, were observed in axons and their Schwann cells, specifically in *Opa1*^**±**^ cochlea (Fig. 3B). Significant reduction in ANF density was observed in *Opa1*^±^ mice from the age of 6 months (*P* ≤ 0.001) compared to 1-month-age mice of the same genotype, and (*P* ≤ 0.05) compared to WT mice of the same age (Fig. 3C). Loss of the ANF density was further increased in *Opa1*^**±**^ mice of 12 months of age (97.1 ± 8.7ANFs per 1 700 µm^2^), while remaining unchanged in WT mice of the same age (12 months: 136.1 ± 7.4, ANFs per 1 700 µm^2^, Fig. 3C). A decrease in ratio of areas (axon area/axon + periaxonal space) was found in *Opa1*^**±**^ mice at 6 months, and becoming significant at 12 months (*P* ≤ 0.05 vs. the age-matched WT mice and vs. 1-month-old *Opa1*^**±**^ mice, Fig. 3D), illustrating an axon retraction. At 6 months, ultrastructural evaluation revealed degenerating axons filled with electron dense inclusions, multiple vacuoles and damaged mitochondria (Fig. 3E–G), thus eventually leading to complete axon degeneration. In addition, *Opa1*^±^ ANF terminals displayed massive increase in abnormal myelin sheaths, with redundant myelin loops and outfoldings (Fig. 3H), split lamellae myelin, and dense myelin debris enclosed within double membrane typical of autophagosomes (Fig. 3I). Additionally, complete degeneration of myelin layers and axons, as well as macrophages containing myelin debris were occasionally observed at the ANF terminals of 12-month-old *Opa1*^±^ mice (Fig. 3J, O), but not in WT animals. Quantification analysis showed an age-related gradual increase in ANF terminals filled with electron dense bodies and multiple vacuoles becoming significant at 6 months, and onwards to 12-monh-old *Opa1*^**±**^ mice, compared to littermates of the same age (*P* ≤ 0.001, Fig. 3K, L). In WT mice, a slight but significant increase (*P* ≤ 0.01, Fig. 3K) of fibers filled with electron dense bodies was only seen at 12 months, together with a significant decrease of axons filled with multiple vacuoles at the same age (*P* ≤ 0.05, Fig. 3L). A significant increase of fibers with irregular myelin features, such as outfoldings and redundant myelin loops, was found in 12-month-old *Opa1*^**±**^ mice (Fig. 3M**)**. Although, a significant increase in degenerating sheaths was observed in 6-month-old *Opa1*^**±**^ mice and maintained to 12 months (*P* ≤ 0.001 vs. 1-month-old *Opa1*^**±**^ or vs. WT mice of the same age, Fig. 3N).

**Fig. 4:**
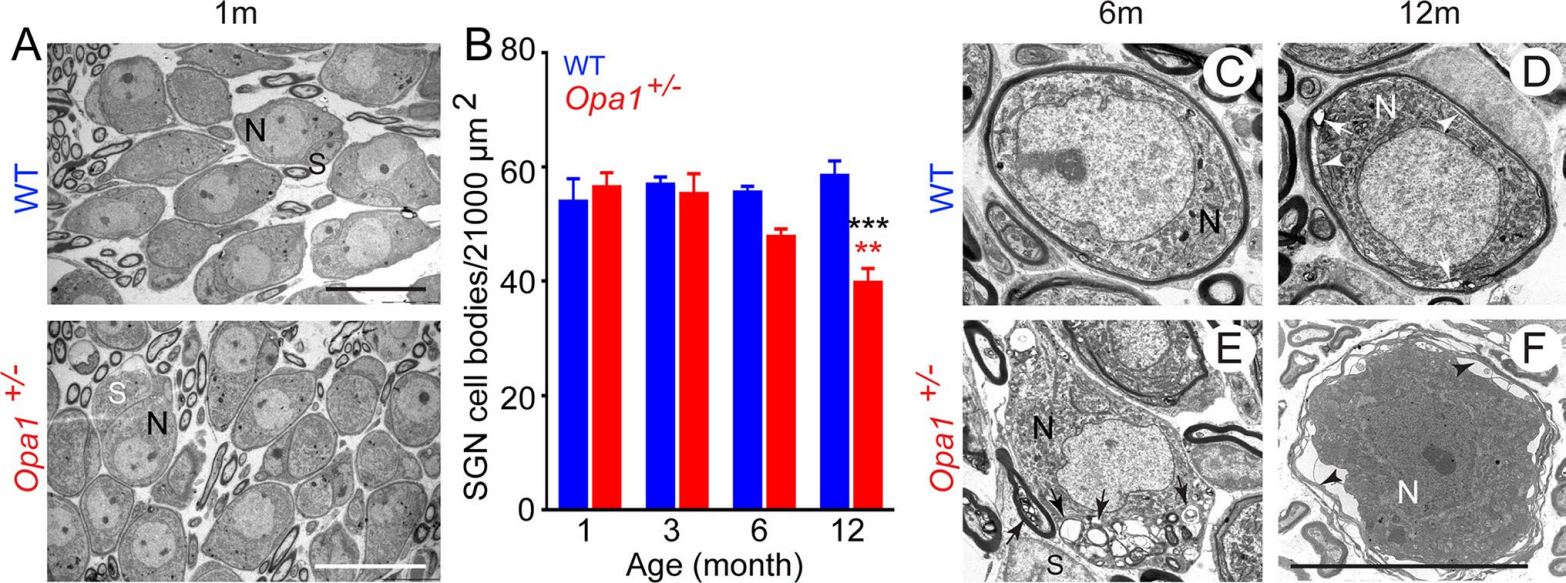
Exacerbated age-related degeneration of spiral ganglion neurons in *Opa1*^±^ mice. **A** Representative transmission electron micrographs of spiral ganglion neurons (SGNs) in WT and *Opa1*^**±**^ mice aged 1 month. **B** Quantitative analysis of SGN density in WT and *Opa1*^**±**^ mice aged 1, 3, 6, and 12 months. All data are expressed as mean ± SEM (*n* = 5 sections per cochlea, 4–5 cochleae per age and genotype). One-way ANOVA test was followed by *Dunn’s* test: *P ≤ 0.05, **P ≤ 0.01, ***P ≤ 0.001. Black asterisks: *Opa1*^**±**^ vs. WT mice of the same age, red asterisks: older *Opa1*^**±**^ vs. 1-month-old *Opa1*^**±**^, blue asterisks: older WT vs. 1-month-old WT. **C–F** Representative transmission electron micrographs of SGN cell bodies (N) and Schwann cells (S) from WT (**C, D**) and *Opa1*^**±**^ aged 6 (**C, E**) and 12 (**D, F**) months. Black and white arrows mark vacuoles (**D, E**) and degenerated myelin sheaths (**F**). Scale bars, **A** = 15 µm, **C–F** = 2.5 µm

Normal morphological appearance and density of SGNs were observed in one-month-old WT and *Opa1*^**±**^ mice (Fig. 4A). A tendency of reduction in the SGN density was observed in 6-month-old *Opa1*^**±**^ mice (Fig. 4B), reaching significance at 12 months, compared to age-matched WT mice (39.75 ± 2.46 vs. 58.50 ± 2.53 for *Opa1*^**±**^ and WT mice*,* respectively), and 1-month-old *Opa1*^**±**^ mice (56.50 ± 2.5, Fig. 4B). In the cochleae of 6-month-old WT mice, the general SGN morphology and the associated glial cells were well preserved (Fig. 4C), while a few small autophagic vacuoles in SGN were only observed in 12-month-old control animals (Fig. 4D).

In 6-month-old *Opa1*^**±**^ mice, autophagic vacuoles were abundant in numerous neurons (Fig. 4E) and glial cell-derived myelin showed non-compacted and split lamellae (Fig. 4E). At 12 months, degenerating SGNs showed shrunken cell bodies with electron dense cytoplasm and nuclei surrounded by degenerating thin and extensive split myelin (Fig. 4F).

Collectively, these results indicate that the abnormality of axons and myelin of ANF terminals is an early event followed by selective IHC loss, which together contribute to the progressive ANSD in *Opa1*^**±**^ mice.

### Differential expression of* Opa1* and other genes in the cochleae of WT and *Opa1*^±^ mice

**Fig. 5:**
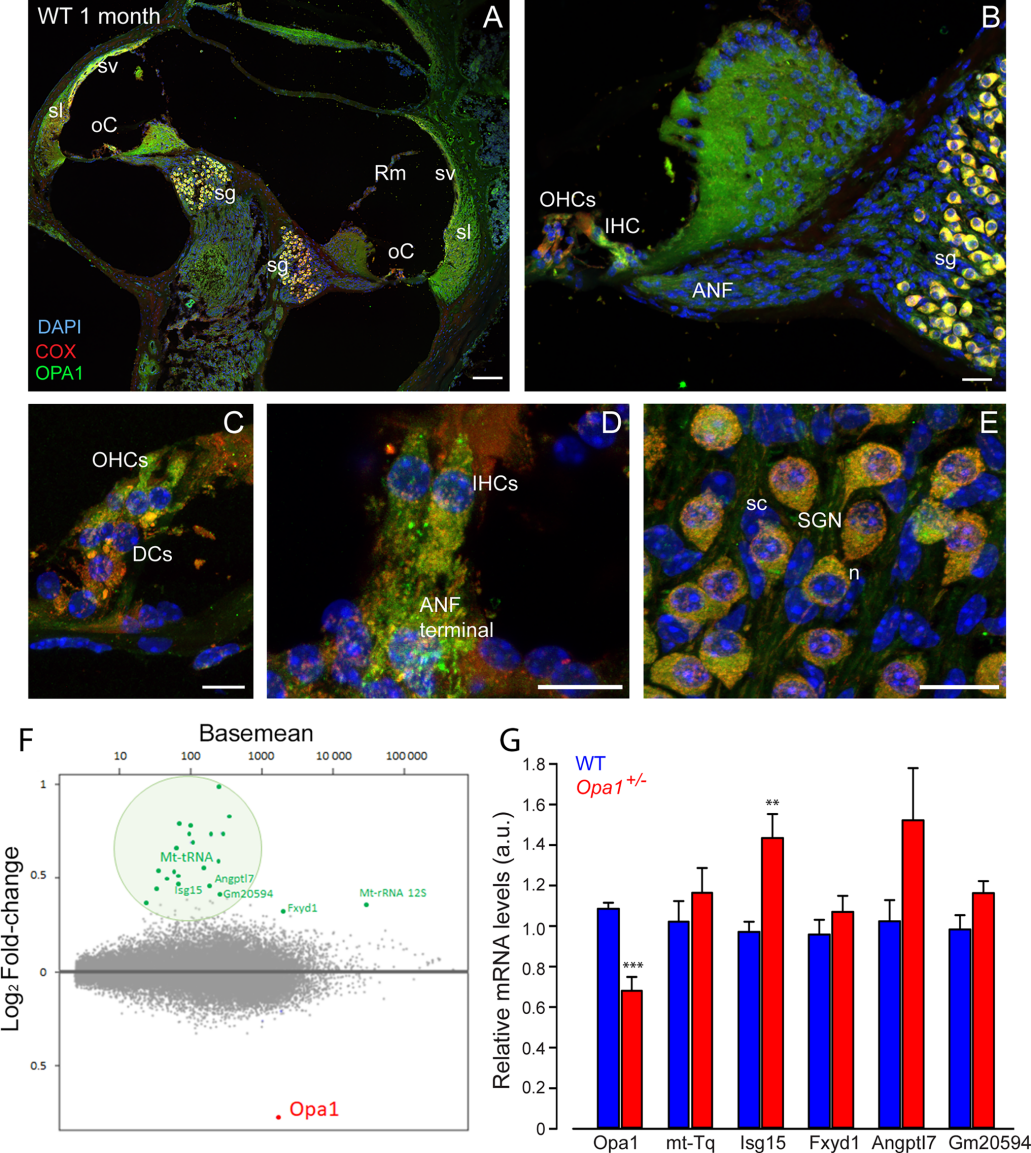
OPA1 expression and mutation induced change in gene expression in the cochlea. **A, B** Confocal images of transverse cryostat sections of a cochlea (**A**) and lower middle turn (**B**) from WT mice at 1 month. Sections were immuno-labeled for COX (red), and OPA1 (green) and counterstained with DAPI to label nuclei. **C–E** Higher magnification of the OHCs (**C**), IHCs (**D**) and spiral ganglion neurons (SGN, **E**). SGN cell bodies (*n*) are surrounded by single Schwann cell (sc). Scale bars = 15 µm. oC: organ of Corti, sv: stria vascularis, Rm: Reissner’s membrane, sl: spiral ligament, ANF: auditory nerve fiber, DCs: Deiters’ cells. All images are representative of *n* = 3–4 cochleae (one cochlea per mouse). **F** Heatmap representing differential gene expression among 15,000 expressed genes (gray dots). Analysis identified a single down-regulated mRNA (Opa1) and 23 up-regulated transcripts. Among these over-represented transcript, 18 correspond to mitochondrial tRNA, 1 to the 12S mitochondrial ribosomal RNA (mt-Rnr1) and 4 to protein-coding mRNA (*n* = 4 *Opa1*^**±**^ mice and 4 control littermates at post-natal p21). **G** RT-qPCR validation of RNA seq data was performed for 5 *Opa1*^±^ and 5 control samples. Although not reaching statistical significance for 4 of 6 differentially expressed genes, the general trend is the same than the one evidenced by RNA seq

To explain the selective susceptibility of IHCs and auditory terminal dendrites, but not other cochlear cells, to *Opa1*^*delTTAG*^ mutation, we investigated cochlear cell localization of Opa1 in 1-month-old WT mice. Immunostaining showed that Opa1 expression was ubiquitously distributed in the cytoplasm of cochlear cells, although the IHCs, OHCs and SGNs displayed higher level of expression than supporting cells (Fig. 5A–E). Double-staining experiments revealed that Opa1 closely colocalizes into the mitochondrial network with the cytochrome c oxidase from the respiratory chain complex IV (Fig. 5A–E).

To explore the pathophysiological mechanism associated to *Opa1*^±^ phenotype, we performed RNA sequencing (RNA seq) analysis of cochlear RNA isolated from 4 *Opa1*^**±**^ mice and 4 control littermates at post-natal p21, before the onset of any adverse phenotype. Differential gene expression analysis identified *Opa1* as the single down-regulated gene, reflecting its haploinsufficiency. Conversely, some RNAs were over-represented, among which the mitochondrial tRNAs and rRNAs (Fig. 5F). We then performed RT-qPCR on a larger group of animals to confirm these RNA seq data. Besides an approximate 40% decrease in *Opa1* mRNA, we disclosed a significant increase in *Isg15* expression, and a trend to increased expression of *mt-Tq, Fxyd1, AngptI7, Gm20594*, in *Opa1*^±^ compared to WT mice (Fig. 5F, G).

Regarding the expression of the 8 *Opa1* mouse splice variants, results from cochlear RNA seq demonstrated that they are transcribed to similar levels in both genotypes (Fig. S3).

### *Opa1*^*±*^ mice show abnormal mitochondrial fragmented ultrastructure and mtDNA deletion

**Fig. 6:**
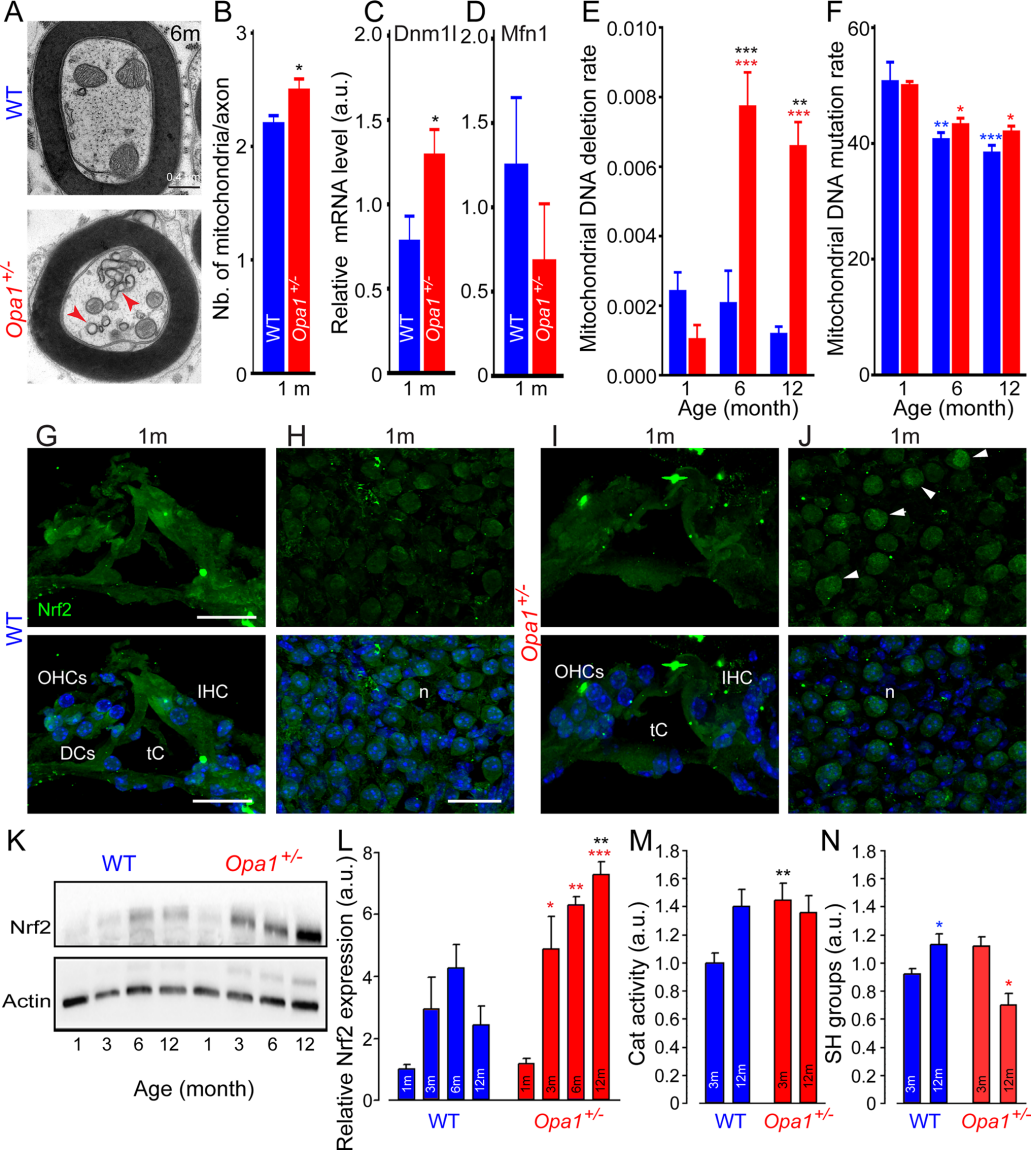
Mitochondrial damage and oxidative stress in *Opa1*^±^ mice. **A** Representative transmission electron micrographs of ANFs from 6-month-old WT and *Opa1*^±^ mice. Scale bar: 0.4 µm. **B** The mean number of the mitochondria per axon from WT and *Opa1*^**±**^ mice at 1 month. **C, D** Quantitative PCR for *Dnm1L* and *Mfn1* transcripts relative to *β-actin* in whole cochlear extracts from WT and *Opa1*^±^ mice aged 1 month. All data are expressed as mean ± SEM (*n* = 8 cochleae per sample). All experiments were performed in technical triplicate. **E, F** Mitochondrial DNA deletion (**C**) and mutation (**C**) rate detected in the cochleae of WT and *Opa1*^**±**^ mice aged 1, 6, and 12 months. Data are expressed as mean ± SEM (*n* = 5 mice per age and genotype). **G–J** Confocal images of transverse cryostat sections of the organ of Corti (**G, I**) and spiral ganglion neurons (**H, J**) from WT (**G, H**) and *Opa1*^**±**^ mice (**I, J**) at 1 months**.** Sections were immunolabeled for Nrf2 (green) and counterstained with DAPI to label nuclei. DCs: Deiters’ cells, tC: tunnel of Corti, n: spiral ganglion neuron. IHCs: inner hair cells, OHCs: outer hair cells. All images are representative of *n* = 4–5 cochleae (one cochlea per mouse) per age and genotype. Scale bars, 15 µm. **K** Representative Western blot analysis for Nrf2 in whole cochlear extracts from WT and *Opa1*^±^ mice. β-Actin is a loading control. **L** Quantification of Nrf2 protein levels in WT and *Opa1*^±^ mouse cochleae. **M, N** Catalase activity (**M**) and SH groups (**N**) in cochlear homogenates from WT and *Opa1*^**±**^ mice. Data are expressed as mean ± SEM (Each experiment was performed with a pool of 8 cochleae per sample per age and per genotype, and in biological and technical triplicate). One-way ANOVA test was followed by *Dunn’s* test: *P ≤ 0.05, **P ≤ 0.01, ***P ≤ 0.001. Black asterisks: *Opa1*^±^ vs. WT mice of the same age, red asterisks: older *Opa1*^±^ vs. 1-month-old *Opa1*^±^, blue asterisks: older WT vs. 1-month-old WT

Transmission electron microscopic examinations of *Opa1*^**±**^ nerve fibers revealed many morphologically abnormal mitochondria, such as fragmented mitochondria with altered cristae and matrix in glia cells as well as in the axoplasm of ANF terminals (Fig. 6A). Measurements revealed a significant increase in the number of mitochondria per fiber in 1-month-old *Opa1*^**±**^ mice compared with age-matched WT mice (*P* ≤ 0.05, Fig. 6B). Interestingly, a significant increase in the *Dnm1L* encoding the dynamin-related protein 1 (Drp-1), which promotes mitochondrial fission, together with a decreased trend of Mfn1 encoding mitofusin-1 (Mfn-1), promoting mitochondrial fusion, were observed in 1-month-old *Opa1*^±^ mice compared with WT mice of the same age (Fig. 6C, D). These results support the fragmentation and consequently the increased number of mitochondria present in the axoplasm.

Because *OPA1* mutations lead to increased mtDNA deletions in patient tissues [11, 47], we analyzed the integrity of the mitochondrial genome in the cochlea. Results from total cochlear mtDNA sequencing revealed significant age-related accumulation of mtDNA deletions in *Opa1*^±^ mice aged 6 and 12 months (*P* ≤ 0.001 vs.1-month-old *Opa1*^**±**^ mice; *P* < 0.01 vs. the age-matched WT mice, Fig. 6E). Conversely, an age-related reduction of the number of mtDNA variants was observed in both strains (6 months vs. 1 month: WT: *P* < 0.01, *Opa1*^**±**^: *P* < 0.05; 12 months vs. 1 month: WT: *P* < 0.001, *Opa1*^**±**^: *P* < 0.05), and no difference between WT and *Opa1*^±^ mice was observed at all age tested (Fig. 6F**).**

Collectively, these results conclusively show that in cochlea, *Opa1*^*delTTAG*^ mutation leads to an imbalance between fission and fusion of mitochondria toward fission, associated to an instability of mtDNA, both processes potentially being responsible for the primary impact on the physiology and further viability of cochlear SGNs and IHCs.

### *Opa1*^±^ mice exhibit activation of Nrf2 pathway and imbalance of redox state

To determine the consequences of *Opa1*^*delTTAG*^ on redox homeostasis in the cochlear tissues, we assessed the activation of Nrf2, a transcription factor accounting for the oxidative stress responses [48]. Our results showed an early nuclear translocation of Nrf2*,* mainly in the SGNs of 1-month-old *Opa1*^**±**^mice compared with WT mice of the same age (Fig. 6G–J). Consistent with this results, a progressive age-related increase in Nrf2 levels was observed in the cochlear tissues of *Opa1*^**±**^ mice from 3 to 12 months of age, but not in WT animals (*P* < 0.05, vs. 1-month age of *Opa1*^**±**^ mice), reaching significance at 12 months (Fig. 6K, L).

Paralleling this discovery, significantly higher level of catalase activity was measured in 3-month-old *Opa1*^**±**^ mice compared with age-matched WT animals (*P* < 0.01, Fig. 6M), and this was maintained until 12 months of age, whereas we uncovered an increase in catalase activity in the cochlear tissues of WT mice only at 12 months (Fig. 6M).

Similarly, measure of thiol groups (SH groups) revealed a significant reduction in the cochleae of *Opa1*^**±**^ mice aged 12 months (*P* < 0.05, Fig. 6N), while this level was significant increase in age-matched WT mice (*P* < 0.05, Fig. 6M). We thus concluded that Opa1 haplo-insufficiency induces Nrf2 activation leading to the upregulation of catalase to counteract oxidative stress.

Upregulation of autophagy

**Fig. 7:**
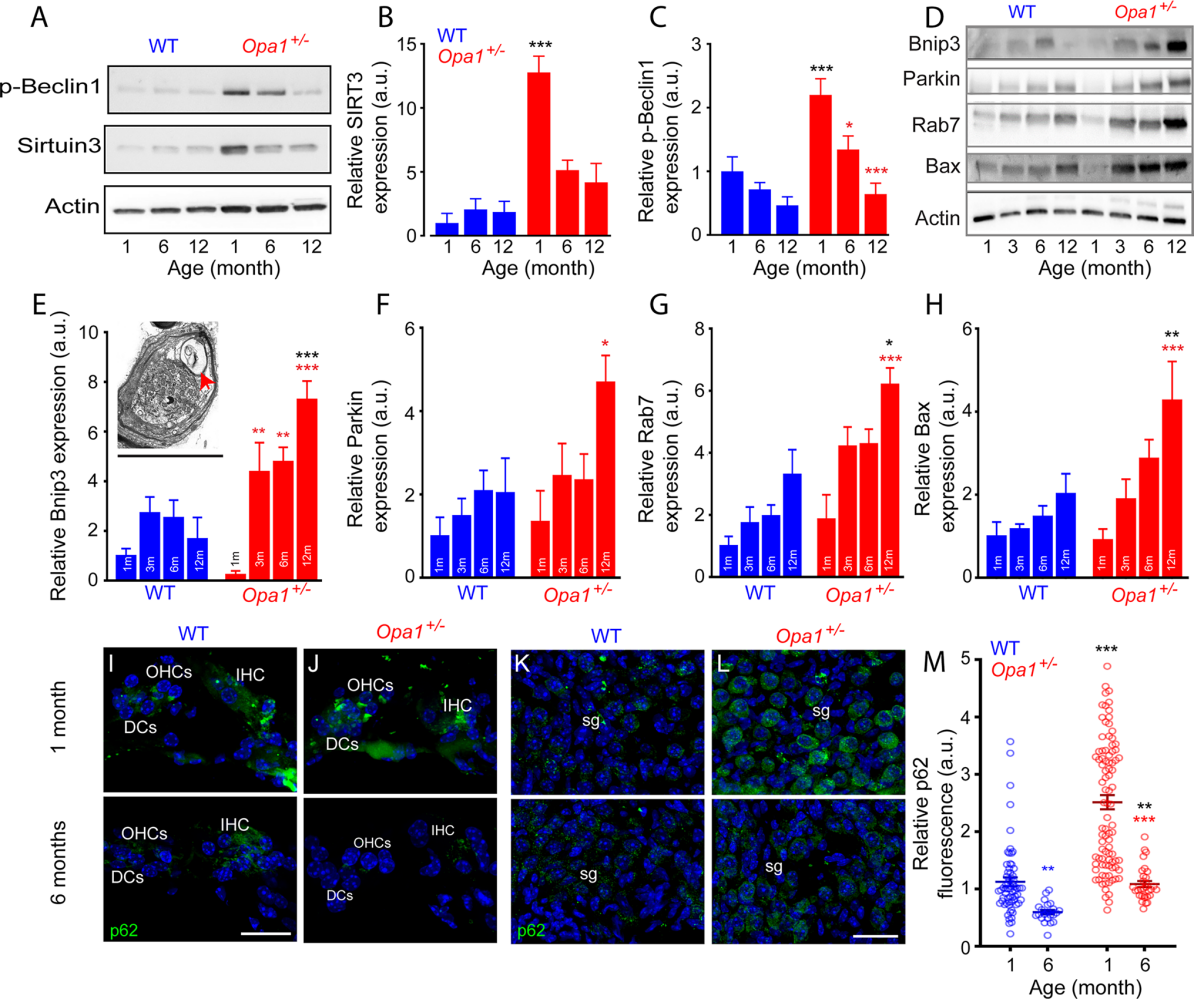
Mitophagy and autophagy. **A** Representative Western blot analysis for phospho-Beclin 1 (p-Beclin1) and SIRT3 in whole cochlear extracts from WT and *Opa1*^±^ mice aged 1, 6, and 12 months. β-Actin is a loading control. **B, C** Quantification of SIRT3 and p-Beclin 1 protein levels in WT and *Opa1*^±^ mouse cochleae. **D** Representative Western blot analysis for Bnip3, Parkin, Rab7, and Bax in whole cochlear extracts from WT and *Opa1*^±^ mice aged 1, 3, 6, and 12 months. β-Actin is a loading control. **Insert** in **E** Representative transmission electron micrograph of SGN from 6-month-old *Opa1*^±^ mice showing a typical autophagosome (red arrow). **E–H** Quantification of Bnip3 (**E**), Parkin (**F**), Rab7 (**G**) and Bax (**H**) protein levels in WT and *Opa1*^±^ mouse cochleae. Data are expressed as mean ± SEM (Each experiment was performed with a pool of 8 cochleae per sample per age and per genotype, and in biological and technical triplicate). One-way ANOVA test was followed by *Dunn’s* test: *P ≤ 0.05, **P ≤ 0.01, ***P ≤ 0.001. Black asterisks: *Opa1*^±^ vs. WT mice of the same age, red asterisks: older *Opa1*^±^ vs. 1-month-old *Opa1*^±^, blue asterisks: older WT vs. 1-month-old WT. **I–L** Confocal images of transverse cryostat sections of the organ of Corti (**I, J**) and spiral ganglion neurons (**K, L**) from WT (**I, K**) and *Opa1*^±^ (**J, L**) mice at 1 and 6 months**.** Sections were immuno-labeled for p62 (green) and counterstained with DAPI to label nuclei. Scale bars, 15 µm. **M** Semi-quantitative analysis of the p62 immunoreactivity in the SGNs of WT and *Opa1*^±^ mice aged 1 and 6 months. All data are expressed as mean ± SEM (*n* = 70 to 90 SGNs from 3 independent cochleae per age and genotype). One-way ANOVA test was followed by *Dunn’s* test (**P ≤ 0.01, ***P ≤ 0.001)

The mitochondrial SIRT3 is a NAD + -dependent enzyme which deacetylates OPA1 to increase its GTPase activity [49]. SIRT3 deacetylates Foxo3, thereby activating the mitophagy through PINK1/Parkin activation [50]. Interestingly, Western blot analyses revealed an early drastic increase in SIRT3 expression in *Opa1*^**±**^ compared to the WT mice at 1 month of age (*P* ≤ 0.001 vs. WT mice of the same age, Fig. 7A, B). Beclin1 is a pro-mitophagic protein with ubiquitous cellular localization [51, 52]. Significant higher level of phospho-Beclin1 (pBeclin1) was also observed in the cochleae of *Opa1*^**±**^ mice aged 1 month (*P* ≤ 0.01, Fig. 7A, C), while decreasing with age in both strains, but only reaching significance in *Opa1*^**±**^ mice (Fig. 7C).

The phosphorylation of Beclin1 together with accumulation of autophagic vacuoles revealed by TEM (Insert Fig. 7E and Fig. 3F, G) led us to exanimate mitophagy and autophagy by measuring the abundances of Bnip3 [53]. We found that *Opa1*^±^ cochleae displayed an age-related increase in Bnip3 in the cochleae of *Opa1*^±^ mice from 3 months and maintained to 12 months (*P* < 0.01, Fig. 7D, E), reaching significance at 12 months (*P* < 0.001, Fig. 7E). We also observed significant age-related increases in parkin, a cytosolic ubiquitin ligase that promotes mitophagy [54] in 12-month-old *Opa1*^±^ mouse cochleae (*P* < 0.05 vs. WT mice of the same age, Fig. 7F) and for Rab7, a small GTP-binding protein involved in the maturation of autophagic vacuoles [55] reaching significance at 12 months in *Opa1*^±^ mice (*P* < 0.001 vs. 1-month age of *Opa1*^±^ mice, *P* < 0.05 vs. age-matched WT mice, Fig. 7G). Altogether, these data strongly suggest that increased mitophagy and autophagic response occurs in the cochleae *Opa1*^±^ mice. In this respect, immunofluorescence experiments showed that similar p62 immunoreactivity was observed in sensory hair cells and the supporting cells of the organ of Corti at 1 and 6 months of age in both strains (Fig. 7I, J), while it was significantly increased in the soma of SGNs of *Opa1*^±^ mice, at 1 month compared to age-matched WT animals (upper panel, Fig. 7K, L and M). Later, significant age-related reduction of p62 immunoreactivity was observed in the SGNs of 6-month-old strains, but with higher levels in the soma of SGNs of *Opa1*^±^ mice compared to WT animals (lower panel, Fig. 7K, L and M), while the abundance of the pro-apoptotic protein Bax increased with age, reaching significance in cochleae of *Opa1*^±^ mice aged 12 months (Fig. 7H).

Collectively, these results suggest that the *Opa1*^*delTTAG*^ allele induces an early increase in mitophagy and autophagy which might reach a threshold impairing the autophagy flux, consequently promoting apoptosis.

## Discussion

*Opa1*^**±**^ mice carry the recurrent *OPA1* c.2708_2711delTTAG variant, recurrently described in DOA patients, which deletes four conserved TTAG base pairs in *Opa1* exon 27. This deletion results in a frame shift, leading to the loss of the last 58 amino acids of all OPA1 isoforms [32]. Here, we demonstrated that the *Opa1*^±^ mice exhibited severe age-related high-frequency sensorineural hearing loss together with a marked reduction in ABR wave-I amplitudes from 6-month age. However, mutant mice harbored larger otoacoustic emissions and similar endocochlear potential in comparison to WT. Ultrastructural examination revealed a selective progressive loss of IHCs and degeneration of the afferent terminals of the spiral ganglion neurons in mutant mice. Together, these results indicate that *Opa1*^*delTTAG*^ mutation induced hearing dysfunction is underlain by ANSD.

### Relevance to human DOA associated auditory neuropathy

Human *OPA1* gene mutations have been associated with DOA, a blinding disease characterized by selective degeneration of retinal ganglion cells and optic nerve atrophy [3, 4, 56]. DOA is the most common inherited optic neuropathy with an incidence of 1:12,000 to 1:50,000 [57]. 20% of mutation carriers, mainly with a missense variant, develop significant neurological deficits including mild-to-moderate sensorineural hearing loss [10, 11, 18]. Despite increasing advances in understanding the pathological mechanisms mediating *OPA1-*related degeneration of retinal ganglion cells, the precise mechanisms underlying the auditory disorders in DOA*plus* remain unknown. Leruez et al., reviewed the files of 1380 patients affected with hereditary optic neuropathies, with 327 patients (24%) harboring OPA1 mutations. Among them, 21 (6.4%) had hearing impairment, revealed by standard pure-tone air and bone conduction audiometry, but only eight patients had been evaluated by speech audiometry, ABR, and DPOAE testing [21]. A few audiological studies of patients harboring the R445H or alternative missense mutations in *OPA1* had an altered function of the terminal unmyelinated portion of the auditory nerve [10, 20, 27]. These results proposed a post synaptic lesion underling an auditory neuropathy [20, 27].

However, the real prevalence of hearing impairments associated with DOA might be higher than reported due to the high variability in disease manifestation and progression [58] as well as the heterogeneity of the clinical profiles of auditory neuropathy [59]. In this respect, it was reported that 25% of patients harboring mitochondrial DNA mutations responsible for Leber’s hereditary optic neuropathy, previously considered as having normal hearing, had electrophysiological evidence of auditory neuropathy [60]. In addition, certain DOA patients might harbor hidden auditory neuropathy, characterized by normal hearing thresholds diagnosed by standard clinical audiometry, but reduced ABR wave-I amplitudes and compound action potential of auditory nerve. These hidden auditory neuropathies are undiagnosed by classic, current clinical audiological tests such as audiogram. In animal models, hidden auditory neuropathy can be caused by moderate noise exposure-, aging-, or genetic factors-inducing a loss of IHC synapses, auditory nerve terminals, and cochlear Schwann cells [59, 61, 62].

Here, we showed that *Opa1 *^*delTTAG*^ mutation caused an ABR threshold shift over time together with progressive reduction in ABR wave-I amplitude and increase in wave-I latency. Meanwhile, DPOAE amplitude was preserved and even enhanced as well as EP was preserved in mutant mice during cochlear aging. The enhancement of DPOAE amplitude observed in mutant mice aged 12 months might result from a decreased inhibitory activity of the medial olivocochlear (MOC) efferents secondary to abnormal auditory nerve fiber activation, as also observed in patients carrying OPA1 mutations [20]. Indeed, OHCs are innervated through numerous direct connections by the MOC efferents [63]. The activation of MOC tends to hyperpolarize OHCs, inhibiting the cochlear amplification.

Our experimental data provide strong evidence that *OPA1*^*delTTAG*^ mutation induces an adult onset and progressive ANSD. These results highlight the need for systematic monitoring of the hearing function including a search for auditory neuropathy in DOA patients using diagnostic tools including pure-tone and speech audiometries, auditory brainstem responses, and optoacoustic emission to finely define both auditory receptor and neural responses to establish the diagnosis of hearing impair in DOA patients. Finally, our results suggest an important role for OPA1 in maintaining IHCs and auditory neural components and hearing against the harmful effects of aging. Interestingly, previous reports have demonstrated that cochlear ribbon synapses are the most susceptible cochlear component to aging insults as well as to noise and ototoxic exposures [62, 64, 65]. Given that cochlear aging often shares the same pathological mechanisms as exposure to noise or ototoxic agents [66], we can assume that *Opa1*^*delTTAG*^ mice are more vulnerable to noise exposures and ototoxic drugs, which needs to be carefully investigated by further studies.

### IHCs and auditory nerve terminals are susceptible to *Opa1*^*delTTAG*^ mutation

Heterozygous mutant mice exhibited a progressive and severe form of age-related deafness that became apparent at 6 months of age and more prominent at 12 months. The delayed and progressive phenotypes suggest mtDNA genome alterations, leading to chronic accumulation of mitochondrial dysfunction and damage, probably in synergy with the deleterious effects of cochlear aging.

The pathophysiological mechanism underlying this hearing disorder pointed to ANSD characterized by selective and progressive loss of IHCs and degeneration of ANF terminals starting from 6 months and becoming prominent at 12 months. Whereas, a similar age-related loss of OHCs was observed in both strains. These results contrast with the preservation, even enhancement of DPOAEs observed in mutant mice during aging. The functional state and morphology of the stria vascularis are also preserved in both strains until 12 months of age. Even though the total number of the ANFs was the same in WT and *Opa1*^±^ mice at 1 month of age, *Opa1*^±^ mice already displayed some anomalies, such as vacuoles, electron dense deposits and damaged remnants of mitochondria in axons and their Schwann cells. By contrast, a later appearance and significant loss of SGNs was observed only at 12 months in mutant mice. Together, these results indicate that IHCs and terminal dendrites are the primary sites of damage. These results are consistent with a previous clinical study on OPA1 syndromic patients suggesting that in addition to the damage of terminal dendrites, inner hair cells could also be affected [20].

The precise mechanisms underlying the selective susceptibility of IHCs and auditory terminal dendrites to *Opa1*^*delTTAG*^ mutation need to be fully disentangled by further in-depth investigations. Our results and previous studies [5, 67] have shown that OPA1 is highly expressed in IHCs, OHCs, vestibular cells, auditory nerve endings, spiral ganglion cells, strial cells, and fibrocytes of the spiral ligament, whereas vestibular dysfunction was reported in only one patient [68], normal or enhanced OHC function was found in DOA patients with OPA1 mutations [20, 26] and in our *Opa1*^±^ mice. Since, the electrochemical environment of the endolymph and endocochlear potential requires normal functioning of the stria vascularis and are important for mechanotransduction of cochlear hair cell as well as for OHC survival [69, 70], normal function of OHCs found in DOA patients assumes that *OPA1* mutations do not affect stria vascularis function. Consistent with these clinical findings, our results revealed an extended preservation of the endocochlear potential and of the stria vascularis morphology, up to 12 months of age in *Opa1*^±^ mice.

The similar results have been observed in the retina of DOA patients or mice carrying *OPA1* mutations. Since OPA1 protein is expressed in all retinal cells, but the selective degeneration is found only in the retinal ganglion cells. Thus, differential tissue expression level of *OPA1* gene or its isoforms does not seem to underlie the selective vulnerability of cochlear IHCs and terminals dendrites as well as retinal ganglion cells to *Opa1* mutations.

One explanation of the selective susceptibility of the retinal ganglion cells and auditory nerve terminal dendrites to *OPA1* mutations might be due to their energy requirements from the initial portion of their axons being unmyelinated [17]. The quantity of mitochondria in unmyelinated auditory nerve afferents is greater for large than small fibers [71], raising the possibility that the hearing disorder linked to *OPA1* mutations may be specific for those auditory nerve fibers rich in mitochondria. Neurons are highly dependent on mitochondria for energy production, and also Ca^2+^ buffering and reactive oxygen species regulation. In addition, the average fiber length between the SGN and the hair cells in humans is about 32 mm [72], which imposes high energy requirements to perform long distance transportation [73]. Energy support by mitochondria along the axon and in SGNs is imperative, thus suggesting the contribution of mitochondrial dysfunction in auditory neuropathy.

To explain the vulnerability of IHCs, but not OHCs to *Opa1*^delTTAG^ mutation, we hypothesize that mitochondrial alterations lead to a reduction in mitochondrial Ca^2+^ buffering capacity [74]. Given that IHCs have a much lower concentration of endogenous calcium buffer in their cytoplasm compared with OHCs [75], IHCs may be less protected than OHCs from the deleterious effects of cytoplasmic Ca^2+^ rise [75, 76].

### OPA1 haploinsufficiency and impaired mitochondrial dynamics are the causal factors of ANSD

Using RNAseq, we clearly demonstrated that the pathophysiological mechanism underlying auditory disorders linked to *Opa1*^*delTTAG*^ mutation is based on Opa1 haploinsufficiency illustrated by the decrease of more than 40% in *Opa1* transcripts in the cochlear tissues of mutant mice at 1 month of age. RNA seq also revealed a coordinated increase in mitochondrial tRNA levels that is a likely to be an indirect consequence of a mitochondrial defect. The number of differentially expressed genes in mutant mice was surprisingly low. We see two possible explanations for this observation: (1) RNA seq analysis was performed on a mixed cell population, an approach that cannot always detect changes in a specific cell type. (2) Unbiased differential analysis of gene expression implies the use of conservative statistical thresholds to avoid false positives and we may have missed minor changes.

It is necessary to note that most patients carrying haplo-insufficient *OPA1* variants display a non-syndromic clinical presentation restricted to a defect of the central visual field [18], whereas the delTTAG variant in mouse *Opa1* gene leading to Opa1 haplo-insufficiency but causes a syndromic presentation [32].

In connection with Opa1 haploinsufficiency, the ANF terminals of mutant mice aged 1 month displayed disorganized mitochondria with missing cristae together with a significant increase in mRNA level of *Dnm1L*, which is a key regulator of mitochondrial fission [77] in the cochlear tissues of mutant mice. Simultaneously, significant increase in the numbers of mitochondria was observed in the terminal dendrites of mutant mice aged 1 month, altogether reflecting impaired mitochondrial dynamics [78]. Indeed, we found that mutant mice are harboring significant increase in mtDNA deletions in the cochlear tissues from the age of 6 months and maintained to 12-month age, thus confirming the role of Opa1 in mtDNA stability in the cochlea. The accumulation of mtDNA deletions in post-mitotic cochlear tissues of *Opa1*^±^ mice during aging might also be explained by an inability to remove these dysfunctional mtDNA copies by mitophagy in the mutant cochleae [22]. These results suggest that mtDNA instability is an event participating to the pathophysiology of age-related hearing impairments related to *Opa1* mutation. The precise mechanisms underlying the selective degeneration of IHCs, auditory nerve fibers, and retinal ganglion cells due to *Opa1* mutation are yet to be fully elucidated. To enhance our comprehension, we propose the incorporation of single-cell RNA sequencing (scRNA-seq) in subsequent studies. Acknowledging the constraints of bulk RNA sequencing employed in our preliminary analyses, we intend to develop a novel Opa1 knock-in mouse model with a GFP reporter selectively expressed in cochlear IHCs and SGNs. Utilizing fluorescence-activated cell sorting (FACS) will enable exact isolation and purification of these specific cells, thus ensuring that the scRNA-seq truly captures the transcriptional profile pertinent to the disease phenotype. Through this precise approach, we aspire to illuminate the intrinsic mechanisms responsible for the cell-specific degeneration related to *Opa1* mutation.

### Oxidative stress, mitophagy, and autophagy control the pathogenicity of *OPA1* mutations in mutant cochlea

In view of the important role of OPA1 in mitochondrial dynamics and maintenance, it is not surprising that Opa1 insufficiency results in an imbalance in mitochondrial dynamics and an increase in fragmented mitochondria and mitophagy shown in our study and others [79]. An interrelationship between impairments in mitochondrial dynamics and increase in ROS generation and oxidative stress has been reported in the cortices of mouse models and patient’s fibroblasts with *Opa1* haploinsufficiency related DOA and in *Caenorhabditis elegans* and *Drosophila melanogaster* carrying mutations in *OPA1* [80, 81]. Together this evidence links OPA1 dysfunction with oxidative stress.

Nrf2 is a redox-sensitive transcription factor and a well-recognized chief regulator of redox homeostasis by triggering the antioxidant enzyme system and regulating both mitochondrial function and biogenesis [82]. Nrf2 has emerged as the main cellular defense mechanism against many harmful environmental toxicants, carcinogens and inducers of neurodegenerative diseases including genetic accelerated age-related hearing loss [48, 83, 84]. Under normal redox conditions, Nrf2 is inactive due to its cytoplasmic retention by Keap1 (Kelch-like ECH-associated protein 1) and rapid degradation by the proteasome system [83]. Under stress condition, Keap1 undergoes modification in its cysteine residues by oxidation or adduct formation. Thus, Keap1 loses its capacity to direct Nrf2 to the ubiquitination complex leading to an increase in Nrf2 levels [85] and its translocation to the nucleus, where Nrf2 induces the expression of a set of antioxidant response element-dependent genes [83, 84, 86, 87]. Here, we found that Nrf2 immunoreactivity is mainly located in the nucleus of SGNs from 1-month-old *Opa1*^±^ mice, suggesting an early nuclear translocation in this cell type, but this process will require further development of technics to specifically isolate SGNs from other cochlear cells and perform semi-quantitative Western blot analyses of Nrf2 levels in the nucleus and cytoplasmic fractions. Interestingly, a significant age-related increase in Nrf2 expression level was also observed in the mutant cochleae from 3 to 12 months. Consistently, a significantly higher level of catalase activity, together with reduced thiol groups were observed in the cochleae ongoing from 3 to 12 months of mutant mice. Our results suggest that *Opa1* haploinsufficiency induces early and persistent activation of Nrf2 in the cochlea to upregulate target genes to counter mitochondrial dysfunction and oxidative stress induced by *Opa1* mutation. Our results are consistent with a previous interesting study showed essential roles of Nrf2 pathway against accelerated age-related hearing loss in *Gjb2*^+/−^ mice carrying a heterozygous human carrier of 35delG [84].

Interestingly, we found that the mutant mouse cochleae displayed a significant early increase of SIRT3, which is a NAD^+^-dependent deacetylase enable to deacetylate and activate OPA1 to enhance mitochondrial fusion [49]. Thus, this early increased SIRT3 expression could generate a compensative mechanism to counterbalance Opa1 haploinsufficiency in cochlear tissues. In addition, an early increase in phospho-Beclin1 was also observed in the cochleae of mutant mice aged 1 month, witnessing early activation of autophagy in the mutant cochleae, since, Beclin1 is a pro-autophagic protein, and its activation through phosphorylation one of the first steps in the assembly of autophagosomes [88].

In addition, more severe age-related increases in mitophagy and autophagy in the mutant cochleae was illustrated by the increased abundance of several mitophagy- and autophagy-related genes and proteins, as well as the accumulation of autophagic vesicles. Finally, the increased level of the autophagic substrate p62, in particular in the SGNs of the mutant cochleae, indicated an impaired autophagic flux [89]. Paralleling this observation, we found that Bnip3, which is a mitochondrial pro-apoptotic BH3-only protein of the BCL2 family interacting with OPA1 [90], is increased in the cochlear tissues during aging.

Collectively, our results demonstrate that an early increase in mitophagy and autophagic response in mutant mice reflect a pro-survival function to remove damaged mitochondria and to respond to auditory cell stress. Later impaired or overloaded autophagic responses may progressively trigger pro-apoptotic stimuli, reaching significant loss of IHC and SGN in the cochleae of old *Opa1*^±^ mice. Finally, the high energy demands of neurons associated with increased clearance of mitochondria along axons and SGNs in the absence of functional OPA1 may disrupt the maintenance of axonal/axon terminals as well as SGNs and result in their degeneration.

## Conclusions

Our data show that *Opa1* c.2708_2711delTTAG mutation in mice leads to an adult-onset progressive ANSD, as attested by selective loss of sensory inner hair cells and progressive degeneration of the axons and the myelin sheaths of the afferent terminals of the spiral ganglion neurons. Molecular investigations revealed that haploinsufficiency of *Opa1* is the disease causing mechanism leading to impaired mitochondrial dynamics, early increased mitophagy and autophagy, and subsequently an age-related increase in mtDNA depletion accumulation, oxidative stress, impaired autophagic flux, and pro-apoptotic death, prompting together the age-related ANSD. These data support a novel role for Opa1 in the maintenance of inner hair cells and auditory neural structures during cochlear aging, and address new challenges for the exploration and hearing rehabilitation of patients with OPA1-linked ANSD.

### Supplementary Information

Below is the link to the electronic supplementary material.

Supplementary file1 (DOCX 861 KB)

Supplementary file2 (XLSX 14 KB)

Supplementary file3 (XLSX 6766 KB)

## Data Availability

All data generated or analyzed during this study are included in this published article and its supplementary information files.

## Abbreviations

ANSD: Auditory neuropathy spectrum disorder
DOA: Dominant optic atrophy
mtDNA: Mitochondrial DNA
DOA*plus*: Dominant optic atrophy plus
ABRs: Auditory brainstem responses
OAEs: Oto-acoustic emissions
OHCs: Outer hair cells
IHCs: Inner hair cells
SGNs: Spiral ganglion neurons
DPOAEs: Distortion product otoacoustic emissions
EP: Endocochlear potential
CAP: Compound action potential
SEM: Scanning electron microscopy
TEM: Transmission electron microscopy
SIRT3: Sirtuin 3
ARHL: Age-related hearing loss
SPL: Sound pressure level
ANFs: Auditory nerve fibers
