# Supplementary material for: The human OPA1delTTAG mutation induces adult onset and progressive auditory neuropathy in mice
Source: Cell Mol Life Sci. 2024 Feb 9;81(1):80. doi: 10.1007/s00018-024-05115-4 (PMC10858076; doi:10.1007/s00018-024-05115-4)
Supplement: Supplementary file 1 — Supplementary file1 (DOCX 861 KB) [file 18_2024_5115_MOESM1_ESM.docx]

**Supplementary Information**

**Additional file 1-2 of The human OPA1^delTTAG^ mutation induces adult onset and progressive auditory neuropathy in mice**

1. **Additional File 1: Figure S1-S3**

**
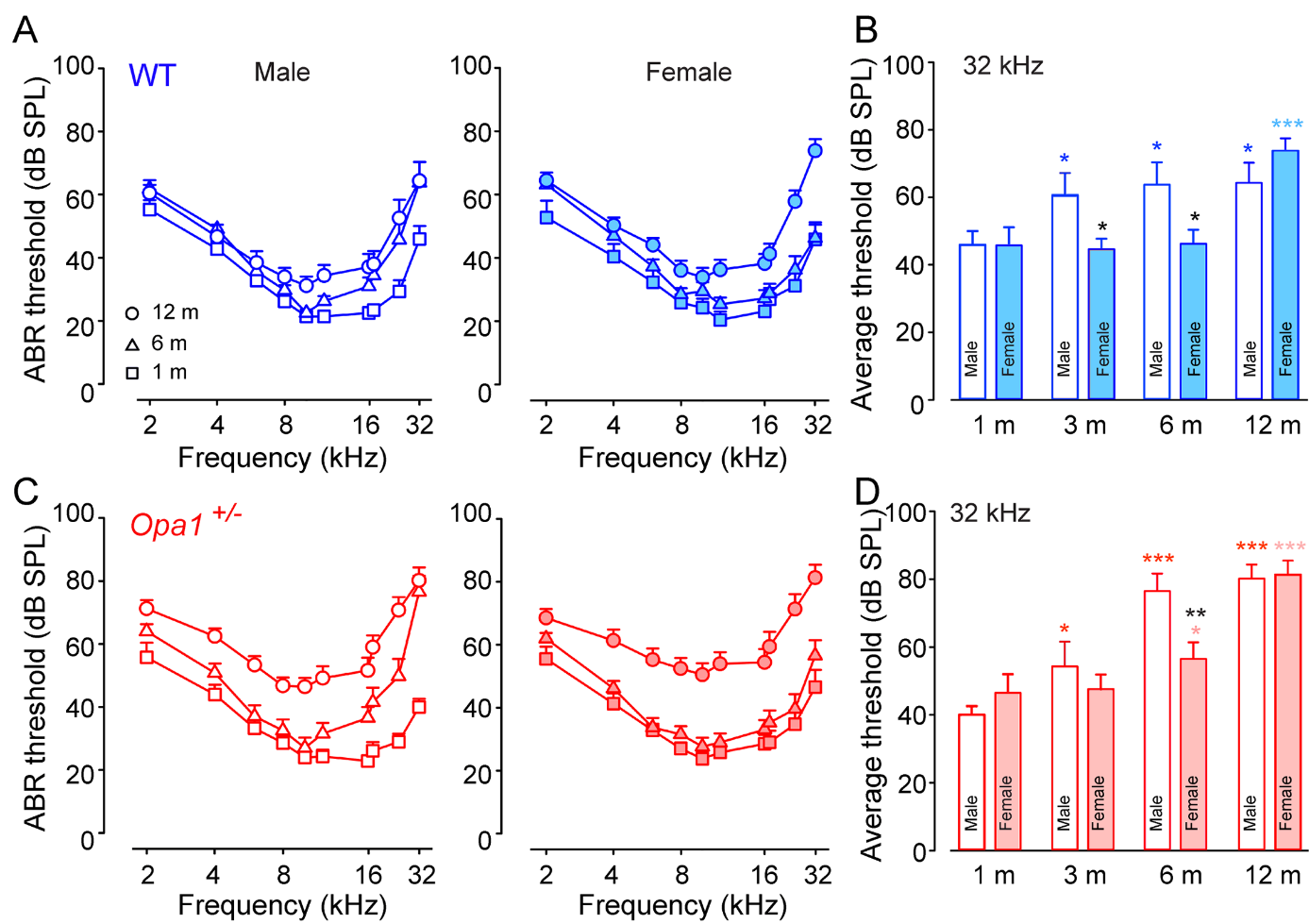
**

**Figure. S1. Exacerbated age-related hearing loss in male and female *Opa1*^+/-^ mice.**

(**A, C**) ABR thresholds recorded in male and female WT and *Opa1***^+/-^** mice aged 1, 6 and 12 months. (**B, D**) Mean ABR thresholds at 32 kHz from WT and *Opa1***^+/-^**  mice aged 1, 3, 6 and 12 months. All data are expressed as mean ± SEM (n=20-40 mice per genotype, sex and time point), one-way ANOVA test was followed by Dunn’s test: **P* ≤ 0.05, ***P* ≤ 0.01, ****P* ≤ 0.001. Black asterisks, female *vs.* male mice of the same age; blue asterisks, older male WT *vs.* 1-month-old male WT; light blue asterisks, older female WT *vs.* 1-month-old female WT; red asterisks, older male *Opa1***^+/-^**  *vs.* 1-month-old male *Opa1***^+/-^**; light red asterisks, older female *Opa1***^+/-^**  *vs.* 1-month-old female *Opa1***^+/-.^**


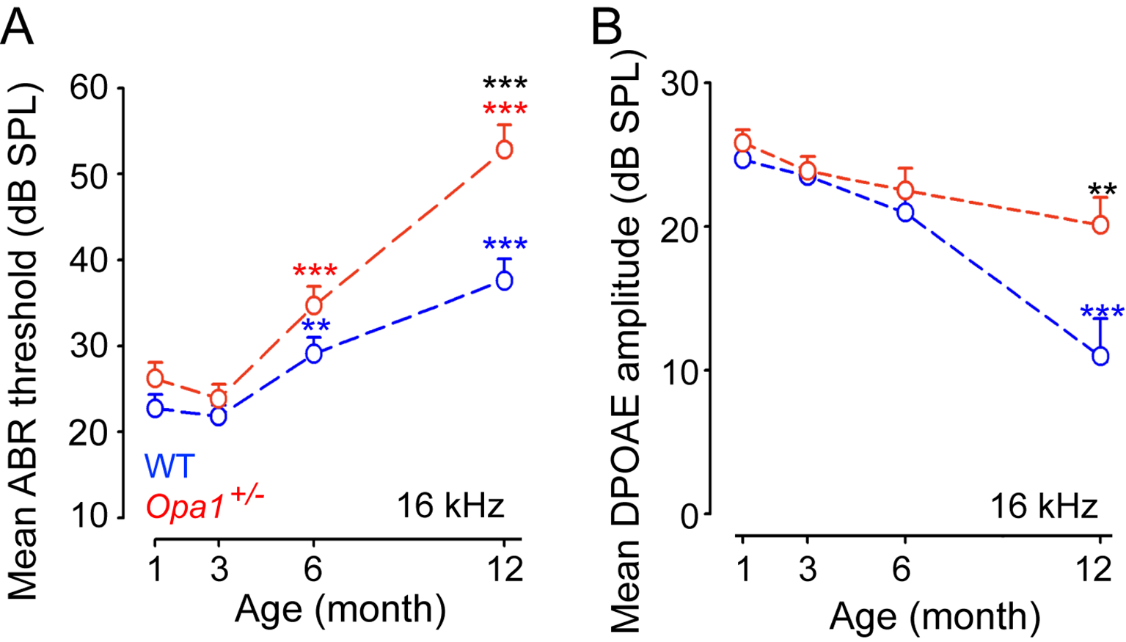


**Fig. S2. *Opa1*^+/-^** **mice exhibit enhanced age-related increase in ABR threshold and reduce in DPOAE amplitude.**

(**A-B**) The mean ABR thresholds (**A**) and DPOAE amplitudes (**B**) at 16 kHz for WT and *Opa1*^+/-^ during age. All data are expressed as mean ± SEM (n=25-40 mice per genotype and time point), one-way ANOVA test was followed by Dunn’s test: **P* ≤ 0.05, ***P* ≤ 0.01, ****P* ≤ 0.001. Black asterisks, *Opa1***^+/-^**  *vs.* WT mice of the same age; red asterisks, older *Opa1***^+/-^** *vs.* 1-month-old *Opa1***^+/-^**; blue asterisks, older WT *vs.* 1-month-old WT (n=25-40 mice per genotype and time point).


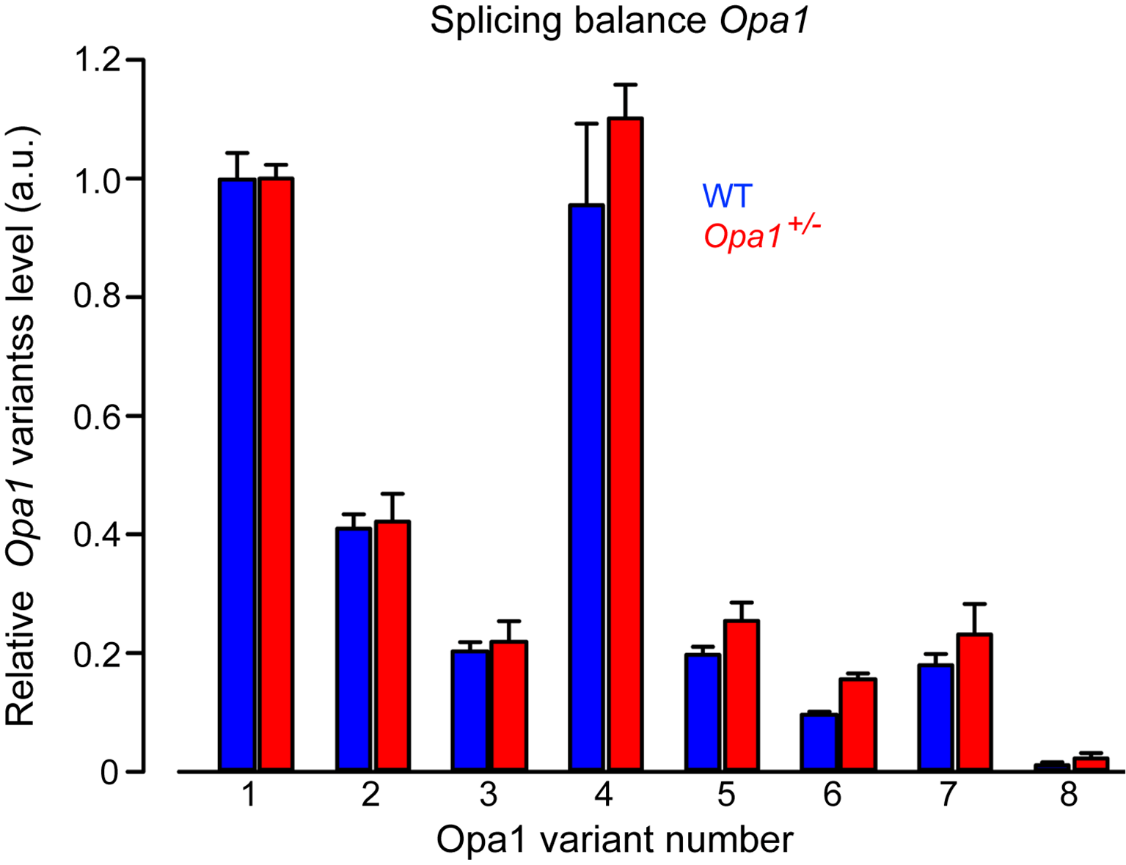


**Fig. S3. The distribution of the 8 *Opa1* mRNAs is not altered in the cochlea of *Opa1^+/-^*mice**

Results from cochlear RNA-seq data were aligned on mouse exons and reads which can be assigned to a single splice variant were collected. Note that eight alternatively spliced Opa1 mRNAs were transcribed (n=4 *Opa1***^+/-^** mice and 4 control littermates).
